# Screening and characterization of biocontrol bacteria isolated from *Ageratum conyzoides* against *Collectotrichum fructicola* causing Chinese plum (*Prunus salicina* Lindl.) anthracnose

**DOI:** 10.3389/fmicb.2023.1296755

**Published:** 2023-12-07

**Authors:** Xiaolin Chen, Miaomiao Zhang, Lihua Tang, Suiping Huang, Tangxun Guo, Qili Li

**Affiliations:** Key Laboratory of Green Prevention and Control on Fruits and Vegetables in South China Ministry of Agriculture and Rural Affairs, Guangxi Key Laboratory of Biology for Crop Diseases and Insect Pests, Institute of Plant Protection, Guangxi Academy of Agricultural Sciences, Nanning, China

**Keywords:** *Bacillus*, biocontrol, antagonistic activity, plum, *Colletotrichum*, volatile

## Abstract

Chinese plum (*Prunus salicina* Lindl.) is a nutritionally and economically important stone fruit widely grown around the world. Anthracnose, caused by *Collectotrichum* spp., is one of the primary biotic stress factors limiting plum production. Medicinal plants may harbor rhizospheric or endophytic microorganisms that produce bioactive metabolites that can be used as anthracnose biocontrol agents. Here, 27 bacterial isolates from the medicinal plant *A. conyzoides* with diverse antagonistic activities against *C. fructicola* were screened. Based on morphological, physiological, biochemical, and molecular characterization, 25 of these isolates belong to different species of genus *Bacillus*, one to *Pseudomonas monsensis*, and one more to *Microbacterium phyllosphaerae*. Eight representative strains showed high biocontrol efficacy against plum anthracnose in a pot experiment. In addition, several *Bacillus* isolates showed a broad spectrum of inhibitory activity against a variety of fungal phytopathogens. Analysis of the volatile organic compound profile of these eight representative strains revealed a total of 47 compounds, most of which were ketones, while the others included alkanes, alkenes, alcohols, pyrazines, and phenols. Overall, this study confirmed the potential value of eight bacterial isolates for development as anthracnose biocontrol agents.

## 1 Introduction

The Chinese plum is one of the most important and delicious fruit crops in the world, grown widely in China, the United States, Japan, and Europe (Wu et al., 2018; Wei et al., 2021). Plum fruits possess high nutritional and economic values, as they are rich in carbohydrates, malic acid, phenolic compounds, anthocyanins, vitamin C, β-carotene, and minerals (Roussos, 2016). Although native to China, the Chinese plum (*Prunus salicina* Lindl.) is also called Japanese plum because the species was first imported into the United States from Japan (Fanning et al., 2014). China is the largest plum producer, with an annual production of 6,615,469 tons in 2021, which accounted for 55.1% of the global total production (Food and Agriculture Organization of the United Nations, 2018).

The production of Chinese plums can be negatively affected by various factors, including a range of diseases (Long et al., 2021; Huang et al., 2022; Shu et al., 2022; Lu et al., 2022a,b). Indeed, the Chinese plum is vulnerable to a variety of diseases, one of the most destructive of which is plum anthracnose, caused by the fungus *Colletotrichum* (Huang et al., 2022). To date, *C. fructicola*, *C. gloeosporioides*, *C. cigarro*, *C. siamense*, and *C*. *aeschynomenes* have been reported as the causative agents of plum anthracnose in China (Huang et al., 2022; Lu et al., 2022a). The pathogen mainly infects the leaves of the plum tree, generally manifesting as yellowish spots or lesions, followed by withering at later stages of the disease. In turn, the resulting reduction in total tree leaf-area limits photosynthesis, nutrient absorption, and growth, ultimately leading to reduced fruit quality and severe yield losses. In addition, plum fruits with visible anthracnose, characterized by sunken, round, and brown necrotic lesions, also experience reduced fruit production at harvest, resulting in considerable economic loss (Hassan et al., 2019).

Most *Colletotrichum* species are plurivorous anthracnose pathogens that infect a wide range of host plants, including many other fruit-tree species besides *Prunus salicina* Lindl (Cannon et al., 2012). To date, anthracnose prevention still relies primarily on chemical control (Wang Q. H. et al., 2020; Wang Z. et al., 2020). Conventional fungicides, such as difenoconazole, prochloraz, tebuconazole, benomyl, and pyraclostrobin are the most common commercial products used to control anthracnose disease in Chinese plum orchards (Dowling et al., 2020; Wang Q. H. et al., 2020; Wang Z. et al., 2020). However, the continuous use of agricultural chemicals directly or indirectly pollutes the air, water, soil, and the overall ecosystem, posing serious health hazards for all living organisms. Therefore, biological control based on the use of microorganisms with antagonistic activities provides a safe and sustainable alternative for controlling phytopathogenic fungi.

Plant-beneficial bacteria that protect plants from disease by living as rhizospheric or endophytic microorganisms, such as *Bacillus*, *Streptomyces*, *Pseudomonas*, and *Burkholderia* have been well documented (Ryu et al., 2014; Boukaew et al., 2017; Guevara-Avendaño et al., 2019; Jin et al., 2019; Soo et al., 2021; Choub et al., 2022; Mehmood et al., 2023; Rodríguez-Cisneros et al., 2023). Some of the bacteria in the genera *Bacillus* and *Pseudomonas* have been demonstrated to have the ability to control anthracnose (Jin et al., 2019; Soo et al., 2021; Choub et al., 2022). Among such antagonistic bacteria, *Bacillus* species are prominent and used as biocontrol agents due to their ubiquitous nature, broad adaptability, and endospore-forming characteristics (Radhakrishnan et al., 2017). Plant-associated antagonistic bacteria have different biocontrol mechanisms against phytopathogens, including competition for resources, production of antimicrobial metabolites, such as antibiotics and lipopeptides, and emission of volatile organic compounds (VOCs), such as ketones and sulfur compounds that can induce plant defense responses and/or antagonize fungal pathogens (Grahovac et al., 2023). For instance, *B. subtilis* controls *C*. *gloeosporioides* by producing antifungal lipopeptides, including iturin A, fengycin, surfactin A, and/or bacillomycin D (Jin et al., 2019). In turn, some rhizobacteria, such as *Pseudomonas* and *Bacillus*, emit VOCs with antifungal activity against *C*. *gloeosporioides* (Guevara-Avendaño et al., 2019; Choub et al., 2022).

Medicinal plants may harbor rhizospheric or endophytic microorganisms that produce bioactive metabolites and may thus be used as biocontrol candidates (Köberl et al., 2013; Aydi Ben Abdallah et al., 2016; Huang et al., 2018; Wicaksono et al., 2018; Momin and Ibrahim, 2020; Zhou A. et al., 2022; Zhou J. et al., 2022). Further, a significant number of bioactive compounds from medicinal plants are now actually known to be produced by associated microbes (Köberl et al., 2013; Huang et al., 2018). A case in point, *Ageratum conyzoides* (Asteraceae) is an annual aromatic herb with great therapeutic importance in many countries worldwide, especially in tropical and subtropical regions. This medicinal plant possesses antifungal potential owning to the secondary metabolites it accumulates against different fungal pathogens, such as *Aspergillus*, *Alternaria*, *Candida*, *Fusarium*, *Phytophthora*, and *Pythium* (Chahal et al., 2021). Additionally, it was previously revealed that endophytic bacteria isolated from *A. conyzoides* exhibited the potential for plant-growth-promoting activities *in vitro* (Momin and Ibrahim, 2020). However, little is known regarding the antimicrobial activity of bacteria isolated from *A. conyzoides*. Therefore, this study aimed to isolate and characterize bacteria from *A. conyzoides*, evaluate their antagonistic activity against plum anthracnose, and confirm their antagonistic activity against a wide range of fungal phytopathogens. In addition, the composition and content of VOCs produced by representative bacterial isolates that showed high antifungal activity in the two-sealed-plate assay were also investigated. Overall, our study confirmed the potential value of eight bacterial isolates obtained from *A. conyzoides* as biocontrol agents.

## 2 Materials and methods

### 2.1 Sampling and microbial strain isolation

*Ageratum conyzoides* samples were collected from Nanning, Hechi, and Yulin cities in the Guangxi Zhuang Autonomous Region, southern China. Flowers, leaves, stems, and roots were selected for the isolation of microbial strains. The *A. conyzoides* sampled organs were cut into small fragments (1 cm in size), sterilized with 75% ethanol for 10 s followed by 2% sodium hypochlorite for 1 min, rinsed three times in sterile water, and homogenized. The samples were allowed to stand for 10 min; then, 100 μL supernatant was diluted to 10^−1^, 10^−2^, and 10^−3^ with sterilized water, and 100 μL of each diluent was spread on LB plates (Supplementary Table S1). After 2 d of incubation at 30°C, different morphotypes were picked up and re-streaked onto LB plates for further purifying a single, pure colony. Subsequently, purified colonies were cultured on LB medium agar (Supplementary Table S1) slants at 4°C and in 25% (v/v) sterile glycerol stock at −80°C for further experiments.

### 2.2 *In vitro* antifungal assay for preliminary screening of antagonistic bacteria

*Colletotrichum fructicola* is the dominant pathogenic species responsible for plum anthracnose (Huang et al., 2022). This species was selected as the preliminary control target- indicator. All bacterial isolates were screened for antagonistic ability using plate confrontation assay (Hong et al., 2022). Generally, the isolated strains were inoculated in LB liquid medium, and cultured on a shaker at 30°C. After cultured for 24 h at 200 rpm, bacterial concentration was adjusted to an OD_600_ value of 1.0. In the plate confrontation assay, the bacterial suspension was streaked using an inoculation loop on the left side of the PDA plate 2 cm away from the edge. A mycelial plug (5 mm in diameter) of each phytopathogenic fungus from the 5-day culture was obtained using a sterile cork borer and placed on a PDA plate at a distance of 5 cm from the center of the inoculated strain line. Plates inoculated with only the phytopathogenic fungal plug served as the control group and were incubated at 28°C for 5 days. Treatments were performed in triplicate. The inhibition rate was calculated using the following formula:

*I* (%) = (*r_0_*–*r_1_*)/*r_0_* × 100

Where *I* is the inhibition rate, *r_0_* is the radius of the mycelia in the control group, *r_1_* is the radius of the mycelia in the dual-culture plate.

Bacterial VOC antifungal activity assays were performed using the two-sealed-plate method (Cortazar-Murillo et al., 2023). Briefly, a 200-μL aliquot of the overnight culture of each bacterial isolate (OD_600_ = 1.0) was spread separately on a plate containing LB medium. The lids were removed and replaced with another PDA plate containing a plug of 5 mm diameter of fungal mycelia on the PDA. Both plates were immediately sealed with a double layer of parafilm to prevent volatile leakage and then incubated at 28°C for 5 days. Each bacterial isolate was tested for antifungal activity in triplicate. Three assays were set up without bacterial treatment (LB only) and used as controls. Antifungal activity was measured as the percentage of reduction in mycelial growth. The inhibition rates were calculated using the following formula:


$$I\left(\%\right)=\frac{d0-d1}{d0}\times 100$$

Where *I* is the inhibition rate, *d*0 is the diameter of mycelial growth in the control, and *d*1 is the diameter of mycelial growth after exposure to bacterial VOCs.

### 2.3 Microscopic observation of the effect of antagonistic bacteria on *Colletotrichum fructicola*

To investigate whether the morphology of *C. fructicola* causing plum anthracnose was altered by the antagonistic bacteria, the mycelia of *C. fructicola* colonies in a 5-day dual culture or in the control medium were observed under a Nikon Ni-E microscope.

### 2.4 Pot experiment to evaluate prevention potential

Potted seedlings of pearl plum, which was developed from the local wild resources of Tian’e County in Guangxi, was used in this experiment. The 2-year-old pearl plum seedlings were transplanted into pots and maintained under uniform field conditions. After growing new leaves, plants in a healthy growth state were selected and prepared for the assays. To prepare bacterial suspensions, each isolate was first grown in LB liquid medium at 30°C in a shaker with 180 rpm for 24 h. All cells were harvested by centrifugation at 5,000 rpm for 5 min and the supernatants were discarded. The pellets were washed, resuspended in 0.05% tween-20 solution, and adjusted to a population density of 1.0 × 10^7^ colony-forming units (CFU)/mL. To obtain the fungal inoculum, after growing on a PDA plate for 5 days, a 5-mm diameter mycelia plug of *C*. *fructicola* was inoculated in PDB liquid medium with a shaking speed of 180 rpm at 28°C in a shaker incubator for 3–4 days. The conidia were collected by filtration and centrifugation, and adjusted to a density of 1.0 × 10^6^ conidia/mL. First, the bacterial suspension was sprayed onto the leaves of the pearl plum seedlings and the control was sprayed with a suspension containing 0.05% tween-20. After the pear plum seedlings were kept in a greenhouse with relative humidity between 75 and 90% for 24 h, 10 μL of conidia suspension was inoculated on each leaf where a round filter paper sheet (0.5 cm in diameter) was placed for moisture. Tree seedlings were inoculated with each strain.

After 5 days, the leaves of the pearl plum trees were examined for symptoms of *C. fructicola* infection and disease severity based on the diameter of necrosis, and disease prevention efficacy was calculated according to the following formula:


$$\mathrm{Pe}\;\left(\%\right)=\frac{d0-d1}{d0}\times 100$$

where Pe is prevention efficacy, *d*0 is the diameter of necrotic lesions when the leaves were inoculated with conidia but without bacterial suspension, and *d*1 is the diameter of necrotic lesions that developed when leaves were inoculated with conidia added with bacterial suspension.

### 2.5 Molecular identification

The antagonistic bacteria were grown in an LB liquid medium at 37°C in a shaker incubator at 180 rpm for 24 h. Genomic DNA was extracted using a TIANamp Bacteria DNA Kit (Tiangen Biochemical Technology Co., Ltd., Beijing, China).16S rDNA, *gyrA*, *gyrB*, and *rpoB* gene fragments of the isolates were amplified using the primers (Roberts et al., 1994; Yamamoto and Harayama, 1995; De Clerck et al., 2004; Wang et al., 2013) listed in Supplementary Table S2. The PCR amplification system consisted of 50 μL of 5 × PCR buffer 10 μL, 4 μL of dNTPs (2.5 mM), 1.5 μL of each primer (10 μM), 0.5 μL DNA polymerase, 1 μL DNA template and adequate ddH_2_O to the final volume of 50 μL. The PCR cycle conditions are listed in Supplementary Table S2, and the PCR products were detected by 1.2% agarose gel electrophoresis. Amplified products were sequenced by Sangon Biotech Co. Ltd. (Shanghai, China). The resulting sequence analysis was performed via BLASTN alignment on NCBI, and the sequences were submitted to GenBank to obtain gene accession numbers. 16S rDNA gene sequences, *gyrA*, *gyrB*, and *rpoB* with high similarity were used to concatenate and construct phylogenetic trees using the maximum likelihood method with bootstrap analysis of 1,000 replicates in the MEGA X software.

### 2.6 Morphological, physiological, and biochemical characterization of antagonistic bacteria

Antagonistic bacterial strains were characterized according to morphological characteristics, such as colony margin, shape, and color, as well as physiological and biochemical characteristics, including Gram reaction, salt tolerance, contact enzyme, V-P test, methyl red (MR) test, citrate utilization, gelatin liquefaction, amylolysis, starch hydrolysis, and carbon source utilization, based on the methods of Berger’s Manual of Bacterial Identification (Holt et al., 1994) and the Common Bacterial Systems Identification Manual (Dong and Cai, 2001).

### 2.7 Evaluation of the antagonistic spectrum of the antagonistic bacteria

The antagonistic spectra of the bacterial isolates against 14 fungal phytopathogens, namely, *Botryosphaeria dothidea*, *Neoscytalidium dimidiatum*, *Pseudofusicoccum violaceum*, *Rhizoctonia solani*, *Exserohilum* sp., *Peronophythora litchi*, *Fusarium sulawesiense*, *Phanerochaete sordida*, *Alternaria brassicae*, *Epicoccum sorghinum*, *Diaporthe phoenicicola*, *D. phaseolorum*, *C. fructicola*, and *Magnaporthe oryzae* were determined by the plate confrontation assay as described above. The phytopathogens were collected and stored in a low-temperature refrigerator at our laboratory.

### 2.8 Effects of different mediums on the antagonistic activity of VOCs

The LB, NA, PSA, and PDA culture media were tested to determine the impact of their composition on the production of VOCs and their involvement in antifungal activity. After growing in PDB, LB, NB, and PSB liquid medium at 30°C in a shaker with 180 rpm for 24 h, 200-μL aliquots of the overnight culture of the isolated bacterial strains were spread on the PDA, LB, NA, and PSA plates, respectively. The two-sealed-plate assay method described above was used for the preliminary assay, and the antagonistic activities of VOCs were compared. The strain with the highest inhibition rate was dual-cultured with *C. fructicola* in two-sealed plates for 24, 48, 72, 96, and 120 h to determine the optimum time for identification.

### 2.9 VOCs identification of bacterial isolates by headspace solid phase microextraction/GC–MS (HS-SPME/GC–MS)

The VOCs emitted by the bacterial isoaltes were identified using headspace solid-phase microextraction coupled with gas chromatography and mass spectrometry (HS-SPME/GC–MS). The following treatment was used to prepare VOCs: each of the eight representative bacterial isolates was grown in LB liquid medium at 30°C in a shaker with 180 rpm for 24 h, then a 200-μL aliquot of the culture was transferred to a headspace vial with 100-mL volume containing 20 mL LB solid medium, and was air-dried on a clean bench. Then, the headspace vial was sealed with a silicone rubber spacer, and placed in a constant temperature incubator at 30°C for 3 days. A headspace vial containing the uninoculated medium was used as a control to remove naturally occurring volatile compounds from each treatment. Subsequently, the headspace vial was placed in a water bath adjusted to 60°C. After equilibration for 5 min, bacterial VOCs were collected with SPME fibers (50/30 μm DVB/CAR/PDMS, Supelco, Inc., Bellefonte, PA), which were inserted into the upper side of the headspace vial for 50 min. Then SPME fibers were injected into the GC port of a gas chromatograph coupled to a mass spectrometer (GC/MS, 7890A-5975CMSD, USA). VOCs were desorbed at 280°C for 5 min in the GC injector port interfaced with a mass detector. Helium gas was used as carrier gas (1.0 mL min^−1^, constant flow) and a DB-5 MS capillary column (30 m length × 0.25 mm inner diameter × 0.25 μm film thickness) from Agilent Technologies (Santa Clara, CA, USA) was used as a stationary phase. Operational conditions were the following: initial oven temperature of 40°C for 5 min, increased to 120°C (4°C per min) for 3 min, and further increased to 280°C (30°C per min) for a run time of 10 min. The mass spectrometer was operated in the electron ionization mode at 70 eV with a source temperature of 250°C, and with a continuous scan from 35 to 450 m/z. Data processing was performed using MassHunter software (Agilent Technologies). VOCs were initially identified by comparing MS peaks with those in the National Institute of Standards and Technology (NIST) 2017 MS Library.

### 2.10 Biocontrol efficacy evaluation under greenhouse conditions

Plum and inoculum preparations were implemented as described in Section 2.4. Two inoculation methods were used in this study. The first method involved spraying the bacterial suspension for 24 h, followed by inoculation with conidia of *C. fructicola* (pre-treatment). In the second procedure, the leaves were inoculated with conidia of *C. fructicola* 24 h before spraying the bacterial suspension (treatment). Three treatments were used: (1) CK, *C. fructicola* alone, (2) *C. fructicola* + antagonistic bacterial isolates, and (3) antagonistic bacterial isolates + *C. fructicola*. Each treatment consisted of three plants with 15 leaves per plant. All pots were kept under greenhouse conditions at a temperature of 28°C and relative humidity between 75 and 90%, under a photoperiod regime of 16 h light /8 h dark.

After 5 days, the leaves of the pearl plum trees were examined for symptoms of *C. fructicola* infection and disease severity (percentage of infected plant area) based on necrosis symptoms. A severity score on a scale of 0 to 5 was based on the visual scale of necrosis on leaves using the modified method of Choub et al. (2021), where 0 = no sign of visible necrosis, 1 = necrotic area covering less than 5% of the total leaf surface, 2 = necrotic lesions covering 6–5% of the total leaf surface, 3 = necrotic lesions covering 16–25% of the total leaf surface, 4 = necrotic lesions covering 26–50% of the total leaf surface, and 5 = necrotic lesions covering more than 50% of the total leaf surface. The incidence rate, disease index, and control efficacy were calculated using the following formulas:


$$\mathrm{Incidence rate}\;\left(\%\right)=\frac{\mathrm{Numer of disease leaves}}{\mathrm{Total number of leaves investigated}}\times 100$$

$$\begin{array}{l} \mathrm{Disease index}\;\left(\%\right)\\ =\frac{\sum \left[\mathrm{Number of disease leaves}\;\mathrm{at}\;\mathrm{each level}\times \mathrm{Corresponding level value}\right]}{\mathrm{Total number of leaves investigated}\times 5}\times 100 \end{array}$$

$$\mathrm{Control efficacy}\;\left(\%\right)=\frac{\mathrm{Disease index in}\;\mathrm{CK}-\mathrm{Disease index in treatment}}{\mathrm{Disease index in}\;\mathrm{CK}}\times 100$$

Software DPS 3.01(Tang and Zhang, 2013) was used for statistical analysis. Statistical significance between data groups was determined at *p* < 0.05 using analysis of variance (ANOV). Data shown are means ± SD (n = 3).

## 3 Results

### 3.1 Isolation of antagonistic bacteria and evaluation of *in vitro* inhibition of *Colletotrichum fructicola*

**Figure 1:**
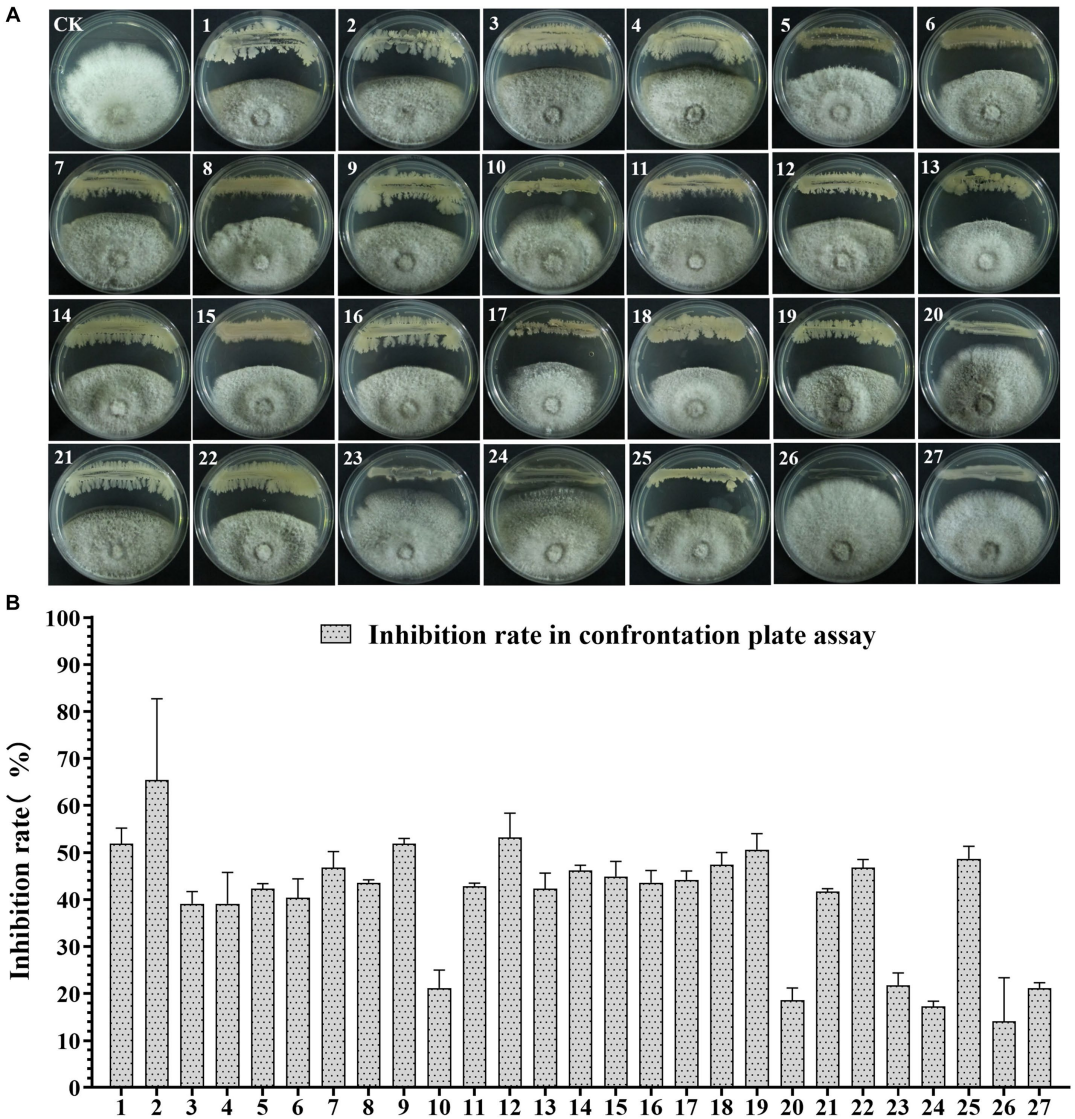
The Antagonistic activity of the 27 isolates against *C. fructicola* in the plate confrontation assay. **(A,B)** CK, negative control; 1, AH7; 2, AH8; 3, AJ4; 4, AJ5; 5, AJ8; 6, AJ9; 7, AY12; 8, AY13; 9, H11; 10, J1; 11, XYDH11; 12, JB4; 13, XYAH1; 14, XYAH2; 15, AYAJ8; 16, XYBH7; 17, XYBJ13; 18, XYCH7; 19, XYCJ12; 20, JB1; 21, XYDJ1; 22, XYDJ2; 23, Y4; 24, Y5; 25, Y6; 26, YB1, 27, H16. Error bars represent the standard deviation (*n* = 3).

**Figure 2:**
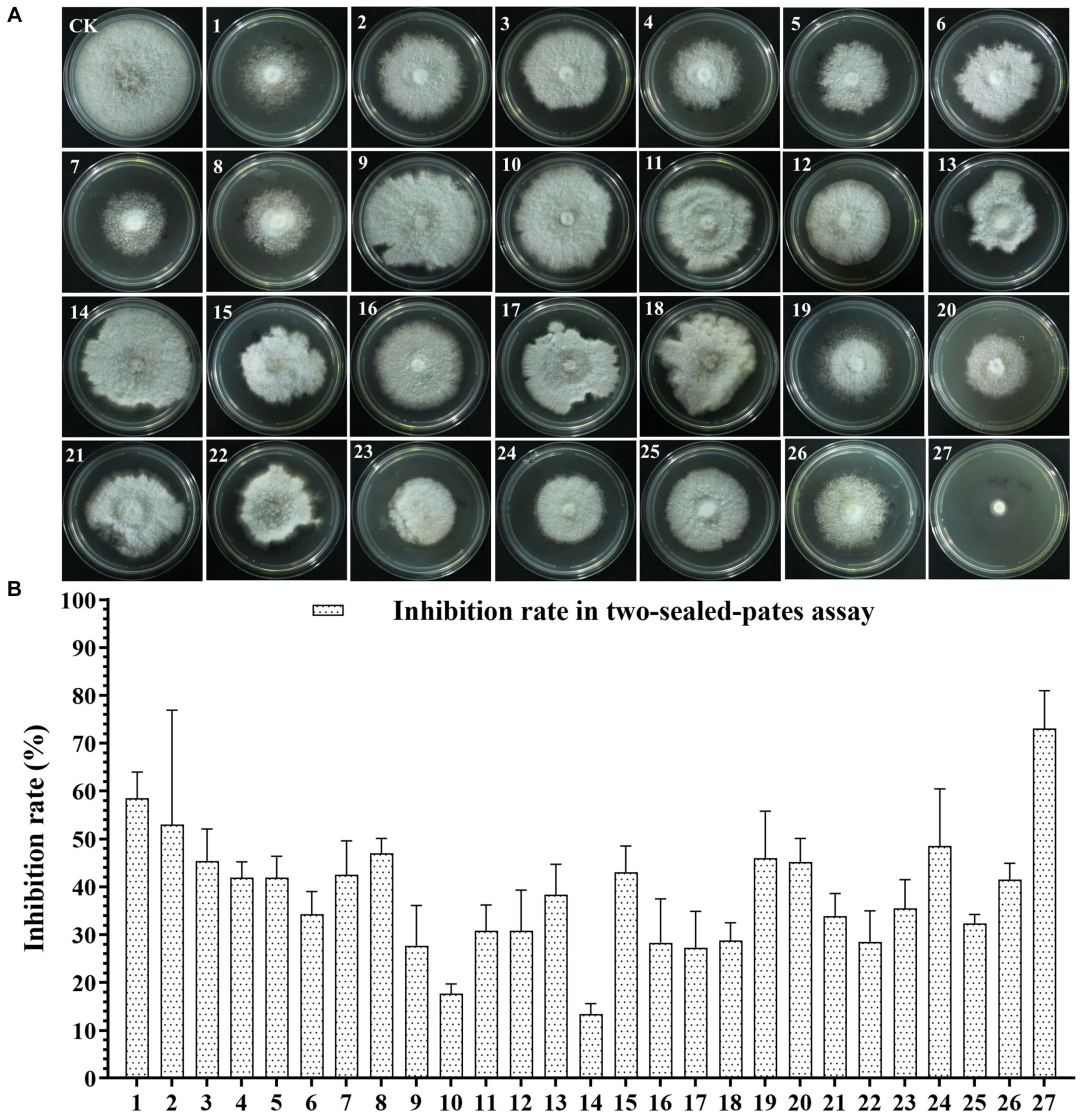
The Antagonistic activity of the 27 isolates against *C. fructicola* in the two-sealed-plate assay. **(A,B)**: CK, negative control; 1, AH7; 2, AH8; 3, AJ4; 4, AJ5; 5, AJ8; 6, AJ9; 7, AY12; 8, AY13; 9, H11; 10, J1; 11, XYDH11; 12, JB4; 13, XYAH1; 14, XYAH2; 15, AYAJ8; 16, XYBH7; 17, XYBJ13; 18, XYCH7; 19, XYCJ12; 20, JB1; 21, XYDJ1; 22, XYDJ2; 23, Y4; 24, Y5; 25, Y6; 26, YB1, 27, H16. Error bars represent the standard deviation (*n* = 3).

**Figure 3:**
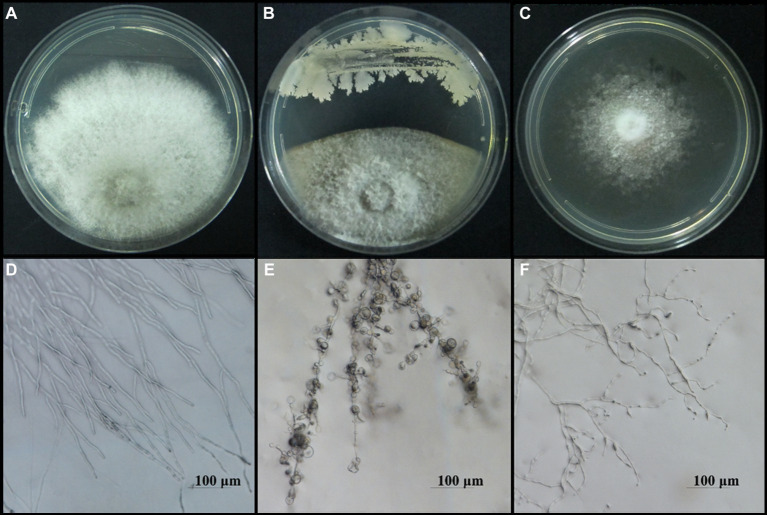
Microscopy images of the morphological alterations in the hyphal structure of *C. fructicola*, causal agent of anthracnose of pearl plum, induced by bacterial isolate AH7. The normal morphology of hyphae in the control groups **(A,B)**, the abnormal hyphae exposed to bacterial diffusible compounds displayed deformation, vacuolation, and swelling **(C,D)**, and hyphae exposed to bacterial VOCs indicated thin, crimped, and shriveling **(E,F)**. Scale bars represent 100 μm.

**Figure 4:**
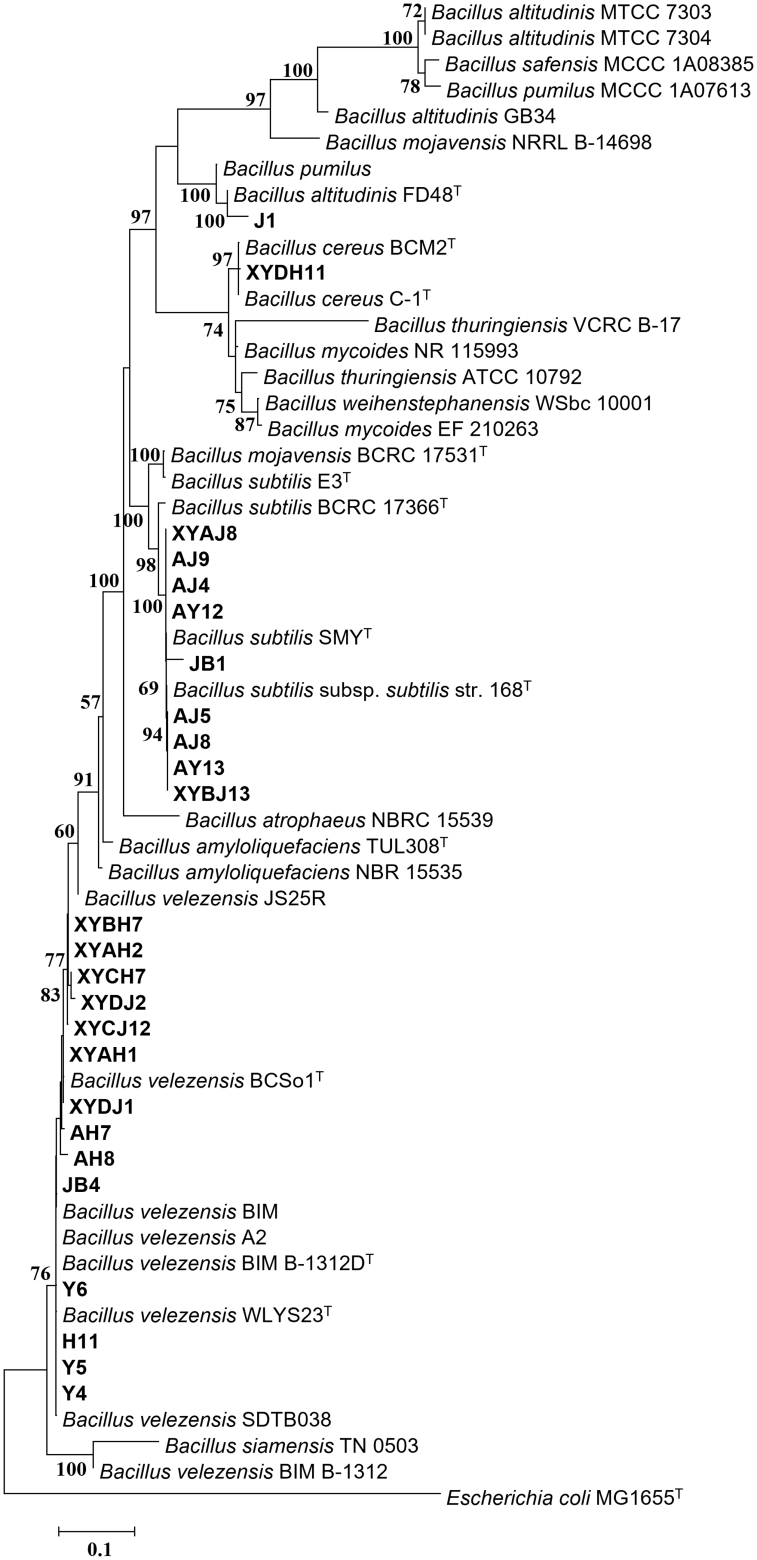
Maximum Likelihood (ML) phylogenetic tree of bacteria strains of *Bacillus* spp. based on 16S rDNA-*gyrA*-*rpoB*-*gyrB* gene sequences. Bootstrap support values (1,000 replicates) above 50% are shown at the nodes. The scale bar shows the evolutionary distance between species.

To screen the microbial resources for controlling pearl plum anthracnose, a total of 249 bacterial strains were isolated from different plant organs (stems, leaves, flowers, and roots) of the *A. conyzoides* collected samples. All isolated bacterial strains were screened for antifungal properties against *C. fructicola* on a dual culture assay. Among them, 27 strains showed varying antagonistic activities; furthermore, particularly, strain AH8 displayed significant antagonism, reaching an inhibition rate of 65.4% in the confrontation plate assay, and strain H16 showed the highest inhibition rate (73.1%) in the two-sealed-plate assay. The inhibition rate of the isolates ranged from 14.1 to 65.4% in the confrontation plate assay and from 13.4 to 73.1% in the two-sealed-plate assay (Figures 1, 2 and Supplementary Table S3). Of the 27 isolates, 9, 6, and 12 were obtained from the flowers, leaves, and stems, respectively (Supplementary Table S4). To determine whether the morphology of *C. fructicola* was changed upon exposure to the antagonistic bacteria, the mycelia at the front line facing the bacterial colony or the medium control were sampled and observed under a microscope. Microscopic observations showed evidence of morphological alterations in fungal mycelia when *C. fructicola* was dual-cultured with the antagonistic bacterial isolate (Figure 3), including severe deformation, swelling, and vacuolation of hyphae exposed to bacterial diffusible compounds (Figure 3D), or thin, crimped, branching, and shriveling of hyphae exposed to bacterial VOCs (Figure 3F), in contrast to hyphae without dual cultured with the isolate developed normally (Figure 4B).

### 3.2 Molecular identification of antagonistic bacteria

**Figure 5:**
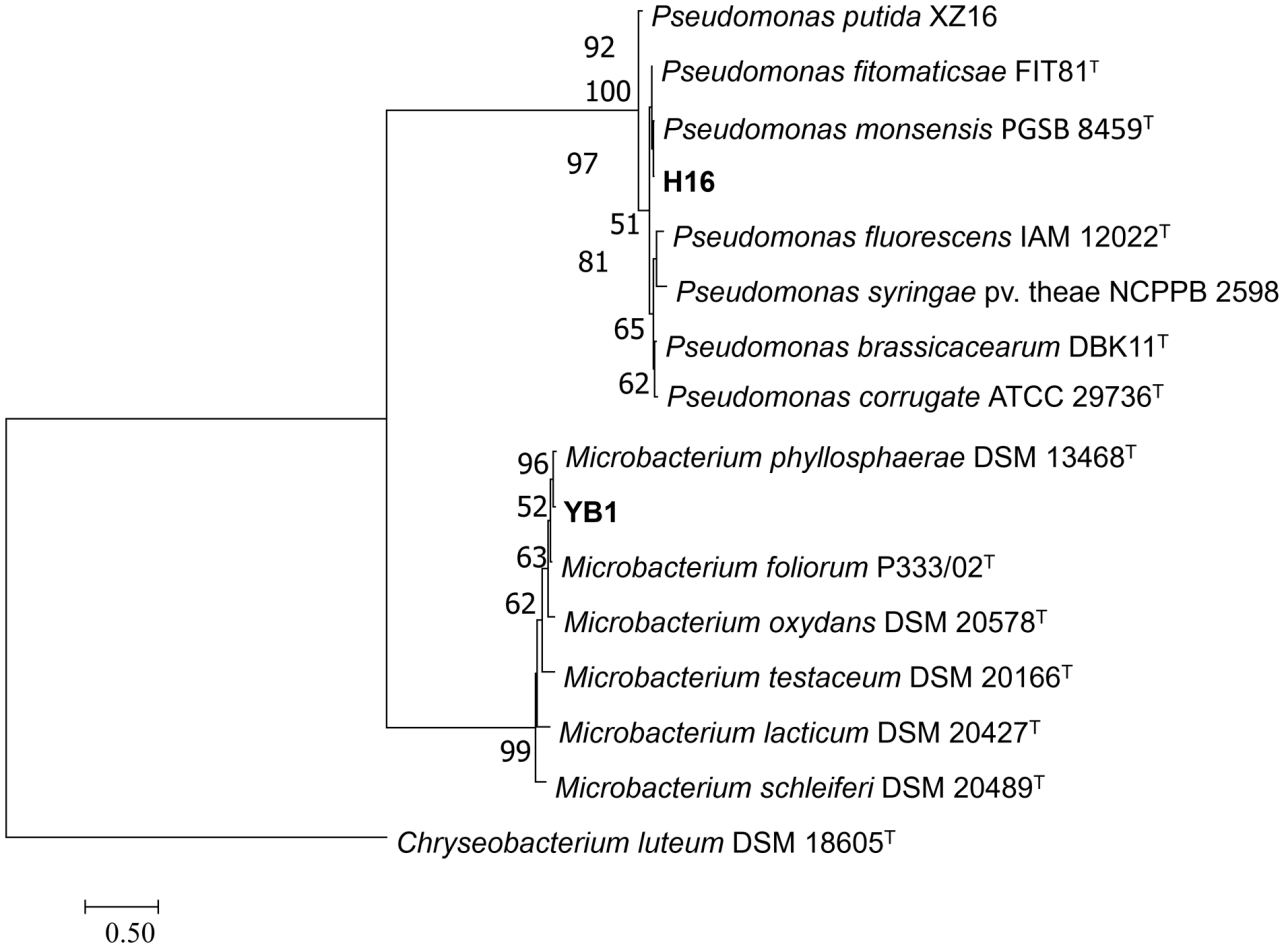
Phylogenetic tree of bacteria strains H16 and YB1 based on 16S rDNA-*gyrB* gene sequences. Bootstrap support values (1,000 replicates) above 50% are shown at the nodes. The scale bar shows the evolutionary distance between species.

To clarify the taxonomic status of the 27 bacterial isolates, 16S rDNA of each isolate was amplified by PCR using the 16S universal primer pair 27F and 1492R. Gene fragments were sequenced and analyzed using BLAST at NCBI. The data showed that 25 isolates had high sequence identity (above 99%) with *Bacillus* spp., and were therefore affiliated with that genus, accounting for 92.6% of the total strains. The two other isolates belonged to genera *Microbacterium* and *Pseudomonas*. Furthermore, *gyrA*, *rpoB,* and *gyrB* from the 25 *Bacillus* strains and *gyrB* from the other 2 strains were amplified and sequenced. All sequences were deposited in GenBank and accession numbers were obtained (Supplementary Table S4). Phylogenetic tree construction based on the concatenated sequences of 16S rDNA, *gyrA*, *rpoB*, and *gyrB* from the 25 isolates and sequences derived from *Bacillus* type strains revealed that 14, 9, 1, and 1 strains were classified as *B. velezensis*, *B. subtilis*, *B. altitudinis*, and *B. cereus*, respectively (Figure 4). Phylogenetic analysis of two other strains, YB1 and H16 based on the 16S rDNA and *gyrB* sequences and sequences derived from the type strains, identified YB1 and H16 as *Microbacterium phyllosphaerae* and *Pseudomonas monsensis*, respectively (Figure 5).

Furthermore, a preliminary evaluation of the prevention of *C. fructicola* damage on pot plants by the 27 antagonistic bacterial isolates showed that most of the bacterial isolates were highly effective in preventing the disease. Among the isolates, XYAH1 and H16 showed the highest prevention efficacy, i.e., 98.4 and 92.0%, respectively (Supplementary Table S5). Therefore, eight representative isolates, *B. velezensis* XYAH1 and XYCJ12, *B. subtilis* XYAJ8 and AJ4, *B*. *creceus* XYDH11, *B. altitudinis* J1, *P*. *monsensis* H16, and *M. phyllosphaerae* YB1, were selected for further experiments.

### 3.3 Morphological, physiological, and biochemical characteristics of antagonistic bacteria

The eight representative isolates were spread on an LB medium plate, placed at 28°C for 2–3 days, and their morphology was observed. Colonies of *Bacillus* spp. were creamy white, opaque, flat, rough, and wrinkled with irregular edges, and there were no obvious differences between them on LB plates (Supplementary Figure S1). A single colony of *P*. *monsensis* H16 was creamy white, round, convex, and smooth, with entire margins. The *M. phyllosphaerae* YB1 colony was beige-to-yellow, round, convex, and smooth, with entire margins (Supplementary Figure S1). Gram staining of *Bacillus* spp. was positive, while *P*. *monsensis* H16 and *M. phyllosphaerae* YB1 were Gram-negative (Supplementary Figure S2). Physiological and biochemical characterization revealed that *Bacillus* spp. grew on LB plates containing 10% NaCl or lower; Meanwhile, *M. phyllosphaerae* YB1 grew on LB plates containing 7% or less NaCk, and *P*. *monsensis* H16 grew on LB plates containing 5% NaCl or less (Supplementary Table S6). There were no apparent differences in the physiological or biochemical characteristics between *B. velezensis* and *B. subtilis*. Both effectively used citrate, sucrose, D-maltose, D-sorbitol, D-xylose, inositol, D-fructose, or D-glucose, but not α-lactose, D-galactose, dulcitol, or L- rhamnose. The contact enzyme, gelatin, starch, cellulose, and V-P reactions were positive, whereas the MR reaction was negative (Supplementary Figure S3 and Supplementary Table S7). *B. cereus* XYDH11 successfully used citrate, sucrose, D-maltose, D-sorbitol, inositol, D-fructose, or D-glucose, but not α-lactose, D-xylose, D-mannitol, D-galactose, dulcitol or L-rhamnose. Other characteristics are the same as *B. velezensis* and *B. subtilis*. Characteristics of *B. altitudinis* J1 are the same as *B. cereus* XYDH11, except MR and V-P reaction, D-maltose, D-sorbitol, inositol, and D-mannitol utilization. With regard to *P*. *monsensis* H16, this strain used citrate, sucrose, D-maltose, inositol, D-mannitol, D-fructose, and D-glucose, but not α-lactose, D-xylose, D-sorbitol, D-galactose, dulcitol or L-rhamnose; other characteristics are the same as *B. cereus* except MR reaction and starch hodrolysis. As for *M. phyllosphaerae* YB1, except starch hydrolysi, α-lactose, D-xylose, D-fructose, and D-glucose utilization other characteristics are the same as *P*. *monsensis* (Supplementary Figure S3 and Supplemental Table S7).

### 3.4 Biocontrol efficacy of antagonistic bacteria against plum anthracnose

**Figure 6:**
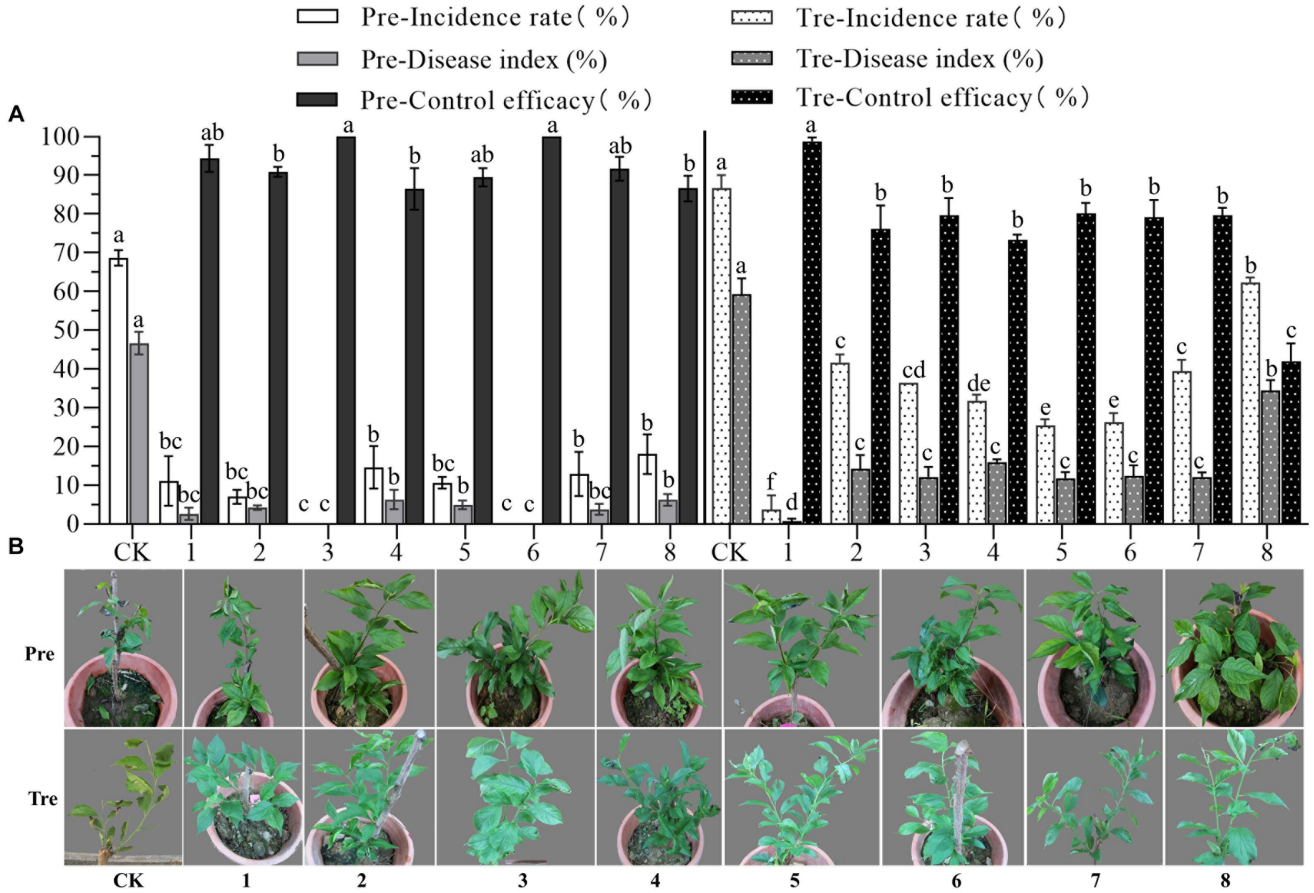
Biocontrol efficacy of the representative bacterial isolates against pearl plum anthracnose under greenhouse conditions. **(A, B)** CK, *C. fructicola* alone; 1, *B. velezensis* XYAH1; 2, *B. velezensis* XYCJ12; 3, *B. subtilis* XYAJ8;4, *B. subtilis* AJ4; 5, *B. receus* XYDH11; 6, *B. altitudinis* J1; 7, *P. monsensis* H16; 8,*M. phyllosphaerae* YB1. Pre: pre-treatment involved spraying bacterial suspension for 24 h first, followed by an inoculation with conidia of *C. fructicola*, Tre, treatment involved inoculating leaves with conidia of *C. fructicola* 24 h before bacterial suspension was sprayed. The photos were taken 5 days post-inoculation.

The biocontrol efficacy of eight representatives against anthracnose of pearl plum was determined under greenhouse conditions. The results showed that all representatives tested had high prevention effectiveness in the pre-treatment, in which case, the prevention efficacy of *B. subtilis* XYAJ8 and *B. altitudinis* J1 reached 100%, and the prevention efficacy of all other bacterial strains was no less than 85%. The disease index and incidence rate of anthracnose on pearl plum leaves in the treatments with the eight tested representatives were significantly lower than those in the control group. Especially were the disease index and incidence rate of leaves in the treatment with *B. velezensis* XYAH1. As a result, in the treatment, *B. velezensis* XYAH1 was the best with a control efficacy of 98.8%; conversely, *M. phyllosphaerae* YB1 showed the poorest control efficacy at 42.0%, whereas the others did not significantly differ in efficacy, ranging from 73.3 to 80.2% (Figure 6 and Supplementary Table S8).

### 3.5 Inhibitory spectrum of antagonistic bacteria

**Figure 7:**
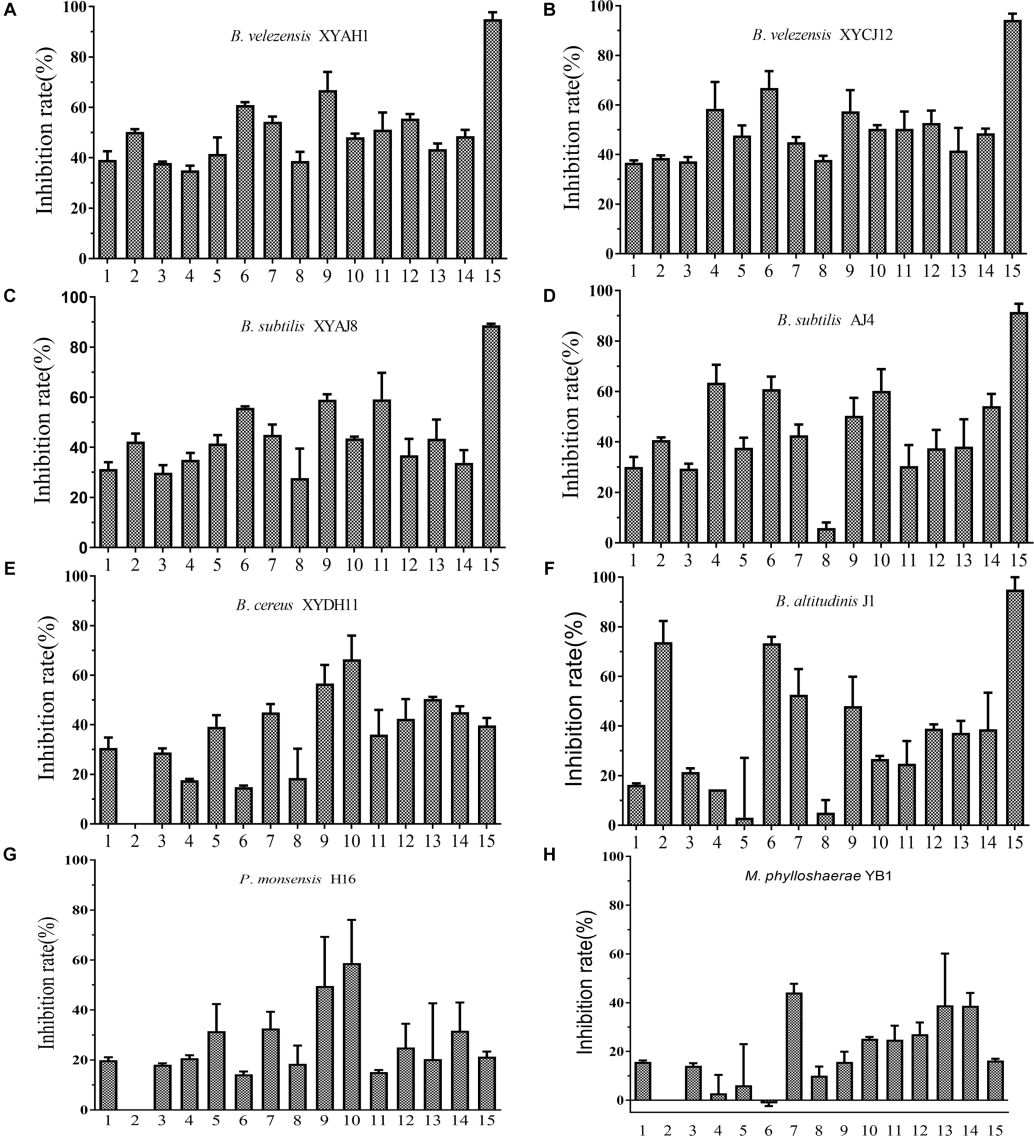
Inhibitory effect of the eight bacterial isolates on 15 phytopathogens. **(A–H)** 1, *Botryosphaeria dothidea* TEY11A-1; 2, *Neoscytalidium dimidiatum* HF; 3, *Pseudofusicoccum violaceum* EGY16A-1; 4, *Rhizoctonia solani* YMWK; 5, *Exserohilum* sp. YB33-1; 6, *Peronophythora litchii* LZ1-3-2; 7, *Fusarium sulawesiense* TEY15-1; 8, *Phanerochaete sordida* FY27-1; 9, *Alternaria brassicae* YB43-2; 10, *Epicoccum sorghinum* YB53-2; 11, *Diaporthe phoenicicola* BY58-2; 12, *Diaporthe phaseolorum* YB3; 13, *Colletotrichum fructicola* DS1-G-1; 14, *C. fructicola* GZ15-1; 15, *Magnaporthe oryzae* 6–6.

The selected eight bacterial strains showed diverse inhibitory activities against the 15 fungal phytopathogens tested (Figure 7). Among the tested bacterial strains, *Bacillus* spp. exhibited relatively better inhibitory effects, whereas *P*. *monsensis* and *M. phyllosphaerae* showed weaker inhibition levels of all tested phytopathogens in the confrontation assay. These results indicated that the *Bacillus* spp. isolates exhibited broad-spectrum antifungal activity. In addition, except *B. cereus* XYDH11, most of the *Bacillus* spp. isolates displayed a high inhibitory effect on *Magnaporthe oryzae*, with inhibition rates ranging from 88.7 to 95.0%, followed by *Peronophythora litchii*, with inhibition rates ranging from 55.8 to 73.4%. However, *B. cereus* XYDH11, *P*. *monsensis* H16, and *M. phyllosphaerae* YB1, had no antagonistic effects on *Neoscytalidium dimidiatum*, and *M. phyllosphaerae* YB1 had no antagonistic effect on *P*. *litchii*.

### 3.6 Characterization of bacterial VOCs using HS-SPME/GC–MS

Volatile compounds produced by the eight bacterial strains *B. velezensis* XYAH1 and XYCJ12, *B. subtilis* XYAJ8 and AJ4, *B*. *creceus* XYDH11, *B. altitudinis* J1, *P*. *monsensis* H16, and *M. phyllosphaerae* YB1, were identified and quantified using GC/MS following headspace solid-phase microextraction (HS-SPME). The data showed that there was no significant difference in the inhibitory effects of strains H16, J1, XYDH11, and XYAJ8 grown in the four different media (Supplementary Table S9). Further, the mean inhibition rate of the eight bacteria grown on LB plates (41.7%) was the highest, whereas the mean inhibition rates on NA, PSA, and PDA plates were 33.5, 31.4, and 33.8%, respectively. Therefore, LB was selected for the analysis of bacterial VOCs. *P*. *monsensis* H16, which showed the highest inhibition rate, was dual-cultured with *C. fructicola* in two-sealed plates for 24, 48, 72, 96, and 120 h, and the results confirmed that 24 h was the best time (data not shown).

Based on the treatment and bacterial volatile-profile analysis, 47 compounds were identified after analyzing the uninoculated sterile medium, and each strain was cultured in the medium using the NIST reference database comparison with similarity above 90% (Supplementary Table S10). The eight selected bacterial isolates with diverse antifungal activity against *C. fructicola* indicated different chemical classes, including ketones, alkanes, alkenes, alcohols, pyrazines, and phenols; however, different strains of the same species did not have the same composition, such as *B. velezensis* XYAH1 and XYCJ12. Additionally, VOCs in the ketone chemical group were present in the volatile profiles of most bacterial isolates and were generally among the most abundant chemical groups (Table 1). The most common VOCs produced by the *Bacillus* isolates were 2-heptanone, 6-methyl-2-heptanone, 4-ethyl-decane, 2-decanone, 2-dodecanone, 2-heptadecanone, 2-heptanone, 2-hexadecanone, 2-nonanone, 2-tridecanone, butylated hydroxytoluene, D-limonene, dibutyl phthalate, and benzyl alcohol. The abundant VOCs methoxy-phenyl-oxime and dimethyl-silanediol produced by *B. subtilis* AJ4, were distinguishable from the VOCs produced by all other *Bacillus* isolates. In *P*. *monsensis* H16, volatile compounds especially ketones, were the most abundant and varied compounds particularly 2-nonanone, 2-undecanone, and tridecanone. In contrast, *M. phyllosphaerae* YB1 mainly contains benzyl alcohol, 4-ethyl-decane, phenylethyl alcohol, and cetene (Supplementary Table S10).

**Table 1:** Main chemical compounds of VOCs produced by bacterial isolates via analysis of HS-SPME/GC–MS.

Bacterial isolates	Compounds	RT (min)	RA (%)	S (%)	Antifungal property	References
*B. subtilis* XYAJ8	**2-heptanone**	**6.00**	**15.45**	**90**	Yes	Sidorova et al. (2023)
	**6-methyl-2-heptanone**	**8.58**	**6.89**	**94**	Yes	Zhang et al. (2022)
	**5-methyl-2-heptanone**	**8.98**	**7.77**	**93**	Yes	Morita et al. (2019)
	D-limonene	11.48	4.42	95	Yes	Wang Q. H. et al. (2020) and Wang Z. et al. (2020)
	Pentacosane	21.49	2.57	91	Yes	Al-Maawali et al. (2023)
*B. subtilis* AJ4	**Dimethyl-silanediol**	**3.79**	**21.15**	**91**	Unknown	–
	**Methoxy-phenyl-oxime**	**8.60**	**44.22**	**90**	Unknown	Barghouthi et al. (2017)
	2-ethyl-3,5-dimethyl-Pyrazine	13.51	1.048	94	Yes	Cherniienko et al. (2022)
	Tetradecane	25.16	1.58	98	Yes	Yuan et al. (2012)
	Pentadecane	28.98	1.29	97	Yes	Yuan et al. (2012)
*B. velezensis* XYAH1	**2-heptanone**	**6.04**	**13.92**	**94**	Yes	Sidorova et al. (2023)
	**6-methyl-2-heptanone**	**8.59**	**25.38**	**95**	Yes	Zhang et al. (2022)
	**2-nonanone**	**14.16**	**4.82**	**95**	Yes	Yuan et al. (2012) and Zheng et al. (2013)
	2-tridecanone	28.70	3.05	96	Yes	Yuan et al. (2012)
	2-tetradecanone	30.75	3.46	91	No	Yuan et al. (2012)
	2-hexadecanone	34.13	3.17	96	Unknown	–
*B. velezensis* XYCJ12	**2-heptanone**	**6.03**	**13.33**	**94**	Yes	Sidorova et al. (2023)
	**6-methyl-2-heptanone**	**8.58**	**8.34**	**95**	Yes	Zhang et al. (2022)
	**2-tridecanone**	**28.71**	**6.96**	**97**	Yes	Yuan et al. (2012)
	2-tetradecanone	30.58	4.74	93	No	Yuan et al. (2012)
	2-hexadecanone	34.13	5.80	95	Unknown	–
*B*. *receus* XYDH11	D-limonene	11.50	2.23	95	Yes	Wang Q. H. et al. (2020) and Wang Z. et al. (2020)
	Benzyl alcohol	11.76	4.62	96	Yes	Zhang et al. (2006)
	**4-ethyl-decane**	**12.94**	**18.81**	**90**	Unknown	–
	**Pentacosane**	**21.21**	**6.21**	**90**	Yes	Al-Maawali et al. (2023)
	**1-decene**	**27.86**	**7.33**	**93**	Unknown	–
*B. altitudinis* J1	**6-methyl-2-heptanone**	**8.60**	**10.54**	**95**	Yes	Zhang et al. (2022)
	**D-limonene**	**11.47**	**3.01**	**95**	Yes	Wang Q. H. et al. (2020) and Wang Z. et al. (2020)
	**4-ethyl-decane**	**13.14**	**9.00**	**90**	Unknown	–
	2-hexadecanone	34.13	2.65	93	Unknown	–
	Dibutyl phthalate	36.45	2.56	96	Yes	Hu et al. (2022)
*P*. *monsensis* H16	**2-nonanone**	**14.24**	**15.67**	**94**	Yes	Yuan et al. (2012) and Sidorova et al. (2023)
	**2-undecanone**	**21.63**	**14.68**	**95**	Yes	Yuan et al. (2012) and Sidorova et al. (2023)
	**2-tridecanone**	**28.82**	**16.55**	**98**	Yes	Yuan et al. (2012)
	2-heptadecanol	29.03	3.68	91	Unknown	–
	2-pentadecanone	33.22	4.67	96	No	Yuan et al. (2012)
*M. phyllosphaerae* YB1	**Benzyl alcohol**	**11.72**	**20.69**	**98**	Yes	Zhang et al. (2006) and Hernández Flores et al. (2022)
	**4-ethyl-decane**	**12.94**	**20.59**	**90**	Unknown	–
	**Phenylethyl alcohol**	**14.73**	**12.0**	**91**	Yes	Xing et al. (2018) and Surovy et al. (2023)
	Benzyl methyl ketone	15.28	2.32	95	Unknown	–
	Cetene	30.55	9.82	95	Yes	Bhat et al. (2020)

Volatile organic compounds were extracted by SPME and identified through GC–MS. Top five of identified compounds in relative area concentration of each isolate and their retention times in GC chromatogram were listed in the table. The compounds with relatively high concentration were in bold text. RT represents the retention times in minutes. RA represents the relative peak area (relative area concentration) of the different compounds detected for each bacterial isolate, expressed as a percentage. S(%), similarity percentage.

## 4 Discussion

Conventional methods for controlling crop diseases mainly rely on the use of chemical pesticides. However, increasing evidence suggests that biological control using beneficial microorganisms (bacteria, fungi, and yeasts) is a safe and promising approach for the management of plant diseases (Liu et al., 2013; Radhakrishnan et al., 2017; Hong et al., 2022; Mehmood et al., 2023). Particularly, endophytic bacteria have been isolated from various medicinal plants, which have potential biological control properties (Shurigin et al., 2021; Tripathi et al., 2022; Kashyap et al., 2023).

Chinese plums are susceptible to a variety of diseases, including anthracnose, leaf spots, brown rot, shoot blight, bacterial shot-holes, and fruit blotching (Long et al., 2021; Huang et al., 2022; Shu et al., 2022; Lu et al., 2022a,b; Lei et al., 2023; Wen et al., 2023; Liu et al., 2023a,b). Anthracnose is one of the most serious diseases resulting in destructive leaf blight in the pearl plum cultivar., in Guangxi, China (Huang et al., 2022). Therefore, in this study, we investigated 249 endophytic bacteria from *A. conyzoides* for their *in vitro* antagonistic activity against *C. fructicola* and screened for potential biocontrol agents to control anthracnose of pearl plum. We found that 27 bacterial strains showed varying antifungal activity levels in both the confrontation plate assay and the two-sealed-plate assay. In addition, culture filtrate of the isolates could also inhibit spore germination of *C. fructicola* completely or partly (data not shown). These findings indicated that the antagonistic properties of these strains may be orchestrated by the emission of soluble and volatile antimicrobial compounds produced by endophytic bacteria with multiple modes of action. Indeed, microscopic observation showed that the morphological alteration in the hyphal structure of *C. fructicola* induced by isolate AH7 resulted from different substances with different modes of action.

Based on physiological and biochemical indicators, as well as molecular characterization, 25 isolates were classified as different *Bacillus* species, including *B. velezensis*, *B. subtilis*, *B. altitudinis,* and *B. cereus*, and two other isolates were identified as *P*. *monsensis* and *M. phyllosphaerae*. Previous studies have demonstrated the potential of many *Bacillus* and *Pseudomonas* species for fungal disease control (Ashwini and Srividya, 2014; Guevara-Avendaño et al., 2019; Jin et al., 2019). For instance, the soil-associated *B. subtilis* strain HN-2 controls *C*. *gloeosporioides* by producing the soluble antifungal lipopeptide bacillomycin D, which injures the cell wall and cell membrane of the hyphae and spores, resulting in the exudation of the cytoplasm and organelles inside the cell (Jin et al., 2019). Some rhizobacteria, such as *Pseudomonas* and *Bacillus*, emitted VOCs with antifungal activity against *C*. *gloeosporioides* (Guevara-Avendaño et al., 2019). Similarly, soil-associated *B. velezensis* CE 100 exhibits antifungal activity against *C*. *gloeosporioides* dependent on various extracellular enzymes, cyclic tetrapeptides, and VOCs that inhibit spore germination and mycelial growth (Choub et al., 2021, 2022). However, endophytic microbes are considered one of the most suitable biocontrol agents because of their high colonization and adaptation potential compared to epiphytic microbes present above or outside the surface of plant tissues (Kashyap et al., 2023).

Walnut trees inoculated with *B. velezensis* CE 100 culture-broth under field conditions showed only 6.5% anthracnose disease severity compared to 8.7% in the conventional treatment group and 45.1% in the control group (Choub et al., 2021). During the pot experiment in our work, pearl plum seedlings inoculated with *B. velezensis* XYAH1 showed a disease index of 0.7% in the treatment group and 59.3% in the control group. In short, the eight representative isolates that were sprayed before inoculating the fungal pathogen *C. fructicola* in the pre-treatment showed highly effective prevention efficacy ranging from 86.5 to 100%. Further, seven isolates that were sprayed after inoculating the pathogen in the treatment also showed high biocontrol potential, with efficacies ranging from 76.1 to 98.8%, except *M. phyllosphaerae* YB1. Thus, our study clearly showed that endophytic bacteria have the potential to be used as effective biocontrol agents against Chinese plum anthracnose. Nonetheless, additional work is needed to determine the feasibility of their application against multiple strains of the pathogen and in real production environments.

In our study, 27 isolates from *A. conyzoides* exhibited diverse antagonistic activities in both the confrontation plate assay and the two-sealed-plate assay. In addition, eight representative isolates showed varying degrees of inhibition against a series of fungal pathogens *in vitro*. Previous studies revealed that endophytes inhibit fungal phytopathogens through several mechanisms: (1) competition for resources, (2) induced systemic resistance, (3) siderophore production, (4) production of soluble antimicrobial compounds, and (5) emission of antimicrobial VOCs (Kashyap et al., 2023). Thus, for instance, the endophytic *B. subtilis* strain 1-L-29 from *Camellia oleifera*, which can produce indole acetic acid, solubilized phosphate, and siderophores, and grow on N-free media, demonstrated antagonistic activity against *C. siamense*, *C*. *asianum*, *Fusarium proliferatum*, *Agaricodochium camellia*, and *P. syringae* (Xu et al., 2020). Similarly, *B. tequilensis* strain YCC 155 from *Crotalaria pallida* has a direct antifungal effect on *C. fructicola* and induces enhanced resistance in *C. oleifera* (Zhou A. et al., 2022). Additionally, *B. velezensis* BR-01, which produces protease, cellulase, β-1,3-glucanase, chitinase, indoleacetic acid, siderophore, and 1-aminocyclopropane-1-carboxylate (ACC) deaminase, etc., shows strong antagonistic activity against a variety of rice pathogens (Zhou J. et al., 2022). In turn, *P. fluorescens* strain BSR2010 from *Bletilla striata* exhibits inhibitory effects against *Staphylococcus aureus*, *Escherichia coli*, *Micrococcus luteus*, and *Pectobacterium carotovorum* subsp. *carotovorum* presumably by producing 2,4-diacetylphloroglucinol (2,4-DAPG) (Wang et al., 2021). These studies indicate that endophytic bacteria isolated from medicinal plants can produce several antimicrobial or bioactive compounds with novel structures. Therefore, using endophytic bacteria present in medicinal plants is of great theoretical and practical value.

Microbial VOCs are particularly important in pathogen suppression and microbial interactions (Tyc et al., 2016). Volatiles have the advantage of easy diffusion in ambient air and in the air contained in soil pores, whereby they can reach farther distances than bacterial soluble compounds in the soil. Previous studies have shown the antagonistic effects of VOCs produced by the genus *Bacillus* against a wide range of fungal pathogens (Guevara-Avendaño et al., 2019). *Pseudomonas* are also capable of emitting antimicrobial VOCs that inhibit the mycelial growth of *Phytophthora infestans*, *Ceratocystis fimbriata,* and *Rhizoctonia solani* (Elkahoui et al., 2015; Zhang et al., 2021; Gfeller et al., 2022). In this study, the VOCs profile of eight representative bacterial isolates were analyzed and the compounds belonged principally to ketones, alkanes, alkenes, alcohols, pyrazines, and phenols (Supplementary Table S10). Many compounds were common to the different isolates. Compared to other VOCs, ketones were produced in particularly high amounts. Most of the compounds showed antifungal activities. A previous study revealed that a *B. amyloliquefaciens* isolate showed antifungal activity against *F*. *oxysporum* f. sp. *cubense*, due to the emission of 2-nonanone, 2-decanone, 2-tridecanone, and 2,3,6-trimethylphenol (Yuan et al., 2012). 2-nonanone and 2-decanone are also emitted by *Bacillus pumilus* and *B. thuringiensis*, and show inhibitory effects on the mycelial growth of *C*. *gloeosporioides* in postharvest mangoes (Zheng et al., 2013). Moreover, 6-methyl-2-heptanone showed inhibited mycelial growth and conidial vitality of *A. solani* (Zhang et al., 2022) and 5-methyl-2-heptanone was considered to be the causative antifungal VOC against *A. alternata*, *Cladosporium cladosporioides*, *Curvularia lunata*, *F*. *oxysporum*, and *Penicillium italicum* (Morita et al., 2019). These studies indicated that the antifungal activity of ketones was negatively correlated with the number of carbon atoms in the ketones. Notably, in this study, 2-nonanone, 2-undecanone, and 2-tridecanone were the most abundant compounds produced by *P*. *monsensis* H16, which significantly inhibited the mycelial growth of *C. fructicola*. The alkane 2-tridecanone and pentacosane might be involved in the suppression of *Alternaria alternata* and other fungi (Al-Maawali et al., 2023). However, the ketone 2-decanone and 2-heptanone, and the alkane 4-ethyl-decane and tetradecane seemed to have no inhibition effects on the mycelial growth of *Rhizopus stolonifer* (Wu et al., 2020). Methoxyphenyl-oxime exhibited a selective antibacterial activity (Barghouthi et al., 2017). Whether this substance possesses antifungal activity needs to be confirmed in further study.

## 5 Conclusion

Twenty-seven bacterial isolates screened for their antagonistic activities *in vitro* and *in vivo* against *C. fructicola* were obtained from the medicinal plant *A. conyzoides* for the first time. Particularly, eight representative strains, including *B. velezensis* XYAH1 and XYCJ12, *B. subtilis* XYAJ8 and AJ4, *B*. *creceus* XYDH11, *B. altitudinis* J1, *P*. *monsensis* H16, and *M. phyllosphaerae* YB1 showed high biocontrol efficacy against Chines plum anthracnose in potted plant experiments, but additional work needs to be done to determine the feasibility of application of them. Further, several *Bacillus* isolates showed broad-spectrum inhibitory activities against a variety of fungal phytopathogens. The VOCs profile analysis of the eight representative bacterial isolates revealed a total of 47 compounds, most of which were determined to be ketone, while others included alkanes, alkenes, alcohols, pyrazines, and phenols. These strains have the potential to be used as biocontrol agents for plant diseases. Integrated use of these strains such as the synthesis of mental nanoparticles using the bacterial metabolites with other control strategies could be a promising alternative method to control Chinese plum anthracnose and other plant diseases. However, further study should be conducted in this aspect.

## Data availability statement

The original contributions presented in the study are included in the article/Supplementary material, further inquiries can be directed to the corresponding author.

## Author contributions

XC: Conceptualization, Data curation, Formal analysis, Investigation, Validation, Writing – original draft. MZ: Data curation, Formal analysis, Validation, Writing – review & editing. LT: Investigation, Writing – review & editing. SH: Investigation, Writing – review & editing. TG: Investigation, Writing – review & editing. QL: Conceptualization, Data curation, Funding acquisition, Investigation, Project administration, Supervision, Writing – review & editing.
