# Supplementary material for: Screening and characterization of biocontrol bacteria isolated from Ageratum conyzoides against Collectotrichum fructicola causing Chinese plum (Prunus salicina Lindl.) anthracnose
Source: Front Microbiol. 2023 Dec 7;14:1296755. doi: 10.3389/fmicb.2023.1296755 (PMC10734640; doi:10.3389/fmicb.2023.1296755)
Supplement: Supplementary file 1 [file Table_1.DOCX]

**Supplemental Figures**


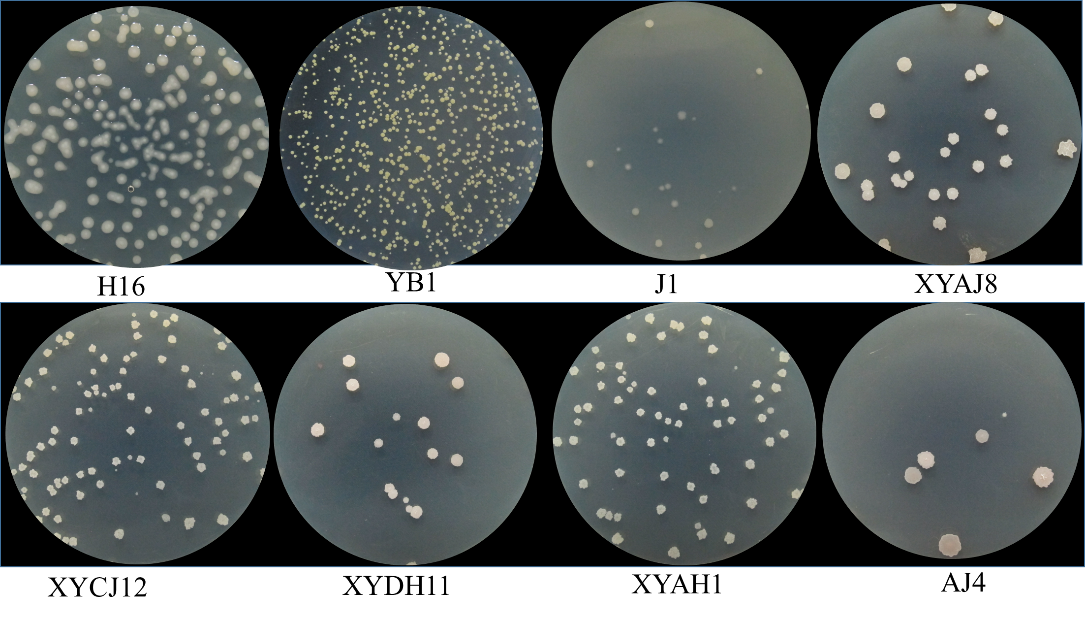


Supplemental Figure 1 Colony morphological characteristics of the eight bacteria


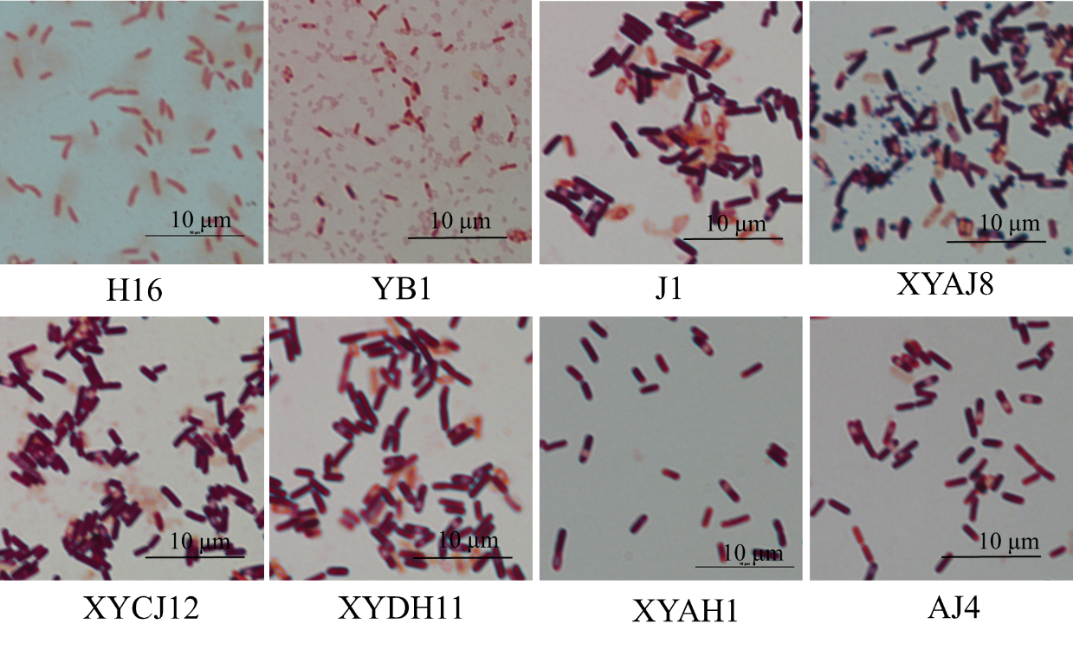


Supplemental Figure 2 Gram staining of the eight bacteria


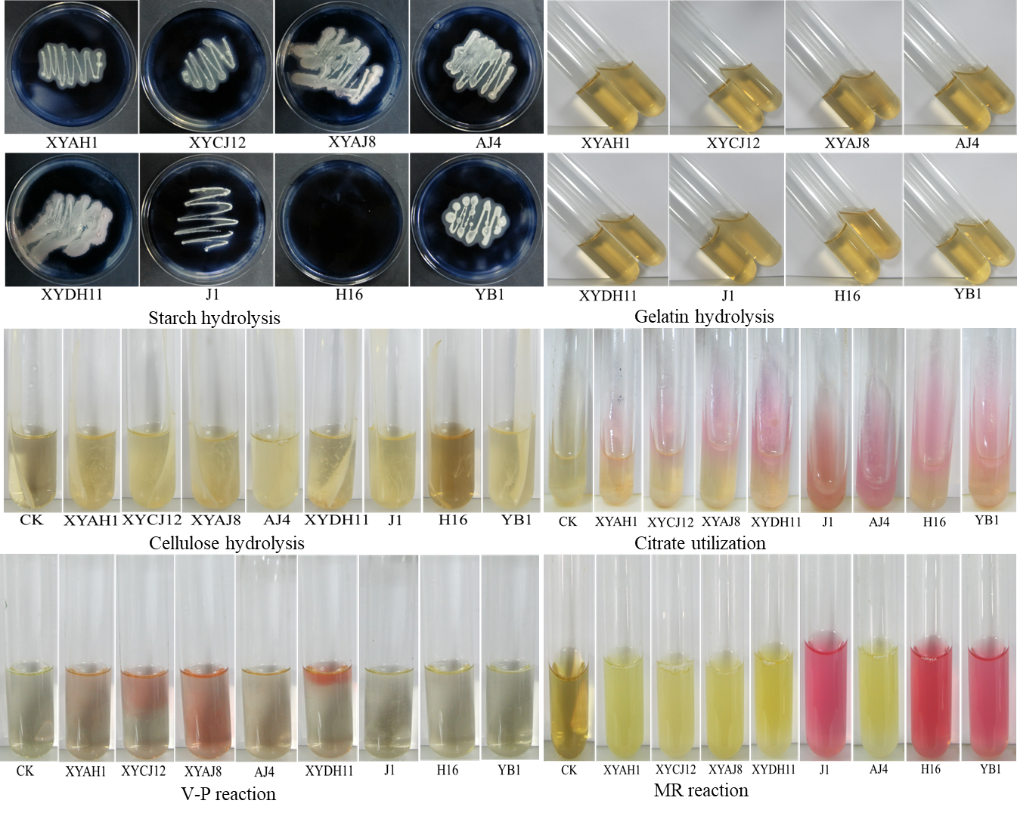


Supplemental Figure 3 Results of physiological and biochemical characterization
