# Supplementary material for: Screening and characterization of biocontrol bacteria isolated from Ageratum conyzoides against Collectotrichum fructicola causing Chinese plum (Prunus salicina Lindl.) anthracnose
Source: Front Microbiol. 2023 Dec 7;14:1296755. doi: 10.3389/fmicb.2023.1296755 (PMC10734640; doi:10.3389/fmicb.2023.1296755)
Supplement: Supplementary file 2 [file Table_2.DOCX]

Supplemental Table 1

Supplemental Table 1 Medium used in this study

| Medium | Formula (per liter) |
| --- | --- |
| PDA | peeled potatoes 200.0 g, glucose 20.0 g, agar 15.0 g, pH7.0 |
| PDB | peeled potatoes 200.0 g, glucose 20.0 g, agar 15.0 g, pH7.0 |
| PSA | peeled potatoes 200.0 g, sucrose 20.0 g, agar 15.0 g, pH7.0 |
| NA | peptone 5.0 g, yeast powder 1.0 g, beef extract 3.0 g, sucrose 10.0 g, agar 15.0 g, pH7.0 |
| NB | peptone 5.0 g, yeast powder 1.0 g, beef extract 3.0 g, sucrose 10.0 g, pH7.0 |
| LA | tryptone 10.0 g, yeast extract 5.0 g, NaCl 10.0 g, agar 15.0 g, pH7.0 |
| LB | tryptone 10.0 g, yeast extract 5.0 g, NaCl 10.0 g, pH7.0 |

Supplemental Table 2 Primers and PCR amplification reaction condition

| Gene | Primer | Sequence (5’–3’) | PCR conditions | Reference |
| --- | --- | --- | --- | --- |
| 16S rDNA | 27F | AGAGTTTGATCCTGGCTCAG | 5 min at 95 °C, 32×(1 min at 94 °C 30 s at 58 °C, 1 min at 72 °C), 10 min at 72 °C | Li et al., 2013 |
|  | 1492R | GGCTACCTTGTTACGACTT |  |  |
| *gryA* | p-gyrA-f | CAGTCAGGAAATGCGTACGTCCTT | 5 min at 95 °C, 30×(1 min at 94 °C, 1 min at 62 °C, 2 min at 72 °C), 10 min at 72 °C | Roberts et al. 1994 |
|  | p-gyrA-r | CAAGGTAATGCTCCA GGCATTGCT |  |  |
| *gyrB* | UP-1 | GAAGTCATCATGACCGTTCTGCAYGCNGGNGGNAARTTYGA | 5 min at 94 °C, 40×(1 min at 94 °C, 1 min at 57 °C, 90 s at 72 °C), 10 min at 72 °C | Yamamoto and Harayama, 1995 |
|  | UP-2r | AGCAGGGTACGGATGTGCGAGCCRTCNACRTCNGCRTCNGTCAT |  |  |
| *rpoB* | RpoB-f | AGGTCAACTAGTTCAGTATGGACG | 5 min at 94°C, 40×(30 s at 94°C, 45 s at 50°C, 2 min at 72 °C), 10 min at 72 °C | De Clerck et al., 2004 |
|  | RpoB-r | ACCGTAACCGGCAACTTAC |  |  |

Supplemental Table 3 The *in vitro* antagonistic activity of the 27 bacterial isolates against against *C*. *fructicola*

| Strain | Inhibition rate in confrontation plate assay (%) | Inhibition rate in bi- plates assay (%) | Strain | Inhibition rate in confrontation plate assay (%) | Inhibition rate in bi- plates assay (%) |
| --- | --- | --- | --- | --- | --- |
| AH7 | 51.9±3.3bc | 58.5±5.5ab | XYAJ8 | 44.9±3.2bc | 43.1±5.4bcde |
| AH8 | 65.4±17.3a | 53.0±23.9abc | XYBH7 | 43.6±2.6bc | 28.3±9.2defg |
| AJ4 | 39.1±2.6cd | 45.4±6.7bcde | XYBJ13 | 44.2±1.9bc | 27.3±7.6defg |
| AJ5 | 39.1±6.7cd | 41.9±3.3bcde | XYCH7 | 47.4±2.6bc | 28.8±3.7defg |
| AJ8 | 42.3±1.1bc | 41.9±4.5bcde | XYCJ12 | 50.6±3.4bc | 46.0±9.8bcde |
| AJ9 | 40.4±4.0bc | 34.3±4.7cdefg | JB1 | 18.6±2.6ef | 45.2±4.9bcde |
| AY12 | 46.8±3.4bc | 42.5±7.1bcde | XYDJ1 | 41.7±0.6bc | 33.9±4.7cdefg |
| AY13 | 43.6±0.6bc | 47.0±3.1bcde | XYDJ2 | 46.8±1.7bc | 28.5±6.5defg |
| H11 | 51.9±1.1bc | 27.7±8.4defg | Y4 | 21.8±2.6de | 35.5±6.0cdef |
| J1 | 21.2±3.8ef | 17.7±2.0fg | Y5 | 17.3±1.1ef | 48.5±12.0bcd |
| XYDH11 | 42.9±0.6bc | 30.8±5.4defg | Y6 | 48.7±2.6bc | 32.4±1.8cdefg |
| JB4 | 53.2±5.2ab | 30.8±8.5defg | YB1 | 14.1±9.3efg | 41.5±3.4bcde |
| XYAH1 | 42.3±3.3bc | 38.4±6.3bcdef | H16 | 21.2±1.1ef | 73.1±7.9a |
| XYAH2 | 46.2±1.1bc | 13.4±2.2g |  |  |  |

Note: Values represent means ± standard error from three replicates, means in each column with the same letters are not significantly different (P < 0.05).

Supplemental Table 4 Information of the 27 isolated strains

| Strain | | Origin | | Accession numbers | | | | | | | | Species | |
| --- | --- | --- | --- | --- | --- | --- | --- | --- | --- | --- | --- | --- | --- |
|  |  |  |  | 16S | | *gyrA* | | *gyrB* | | *rpoB* | |  |  |
| AH7 | | flower | | OQ382856 | | OQ408160 | | OQ408185 | | OQ408210 | | *Bacillus velezensis* | |
| AH8 | | flower | | OQ382857 | | OQ408161 | | OQ408186 | | OQ408211 | | *B*. *velezensis* | |
| AJ4 | | stem | | OQ382858 | | OQ408162 | | OQ408187 | | OQ408212 | | *B*. *subtilis* | |
| AJ5 | | stem | | OQ382859 | | OQ408163 | | OQ408188 | | OQ408213 | | *B*. *subtilis* | |
| AJ8 | | stem | | OQ382860 | | OQ408164 | | OQ408189 | | OQ408214 | | *B*. *subtilis* | |
| AJ9 | | stem | | OQ382861 | | OQ408165 | | OQ408190 | | OQ408215 | | *B*. *subtilis* | |
| AY12 | | leaf | | OQ382862 | | OQ408166 | | OQ408191 | | OQ408216 | | *B*. *subtilis* | |
| AY13 | | leaf | | OQ382863 | | OQ408167 | | OQ408192 | | OQ408217 | | *B*. *subtilis* | |
| H11 | | flower | | OQ382864 | | OQ408168 | | OQ408193 | | OQ408218 | | *B*. *velezensis* | |
| J1 | | stem | | OQ382865 | | OQ408169 | | OQ408194 | | OQ408219 | | *B*. *altitudinis* | |
| XYDH11 | | flower | | OQ382866 | | OQ408170 | | OQ408195 | | OQ408220 | | *B*. *cereus* | |
| JB4 | | stem | | OQ382867 | | OQ408171 | | OQ408196 | | OQ408221 | | *B*. *velezensis* | |
| XYAH1 | | flower | | OQ382868 | | OQ408172 | | OQ408197 | | OQ408222 | | *B*. *velezensis* | |
| XYAH2 | | flower | | OQ382869 | | OQ408173 | | OQ408198 | | OQ408223 | | *B*. *velezensis* | |
| XYAJ8 | | stem | | OQ382870 | | OQ408174 | | OQ408199 | | OQ408224 | | *B*. *subtilis* | |
| XYBH7 | | flower | | OQ382871 | | OQ408175 | | OQ408200 | | OQ408225 | | *B*. *velezensis* | |
| XYBJ13 | | stem | | OQ382872 | | OQ408176 | | OQ408201 | | OQ408226 | | *B*. *subtilis* | |
| XYCH7 | | flower | | OQ382873 | | OQ408177 | | OQ408202 | | OQ408227 | | *B*. *velezensis* | |
| XYCJ12 | | stem | | OQ382874 | | OQ408178 | | OQ408203 | | OQ408228 | | *B*. *velezensis* | |
| JB1 | | stem | | OQ382875 | | OQ408179 | | OQ408204 | | OQ408229 | | *B*. *subtilis* | |
| XYDJ1 | | stem | | OQ382876 | | OQ408180 | | OQ408205 | | OQ408230 | | *B*. *velezensis* | |
| XYDJ2 | | stem | | OQ382877 | | OQ408181 | | OQ408206 | | OQ408231 | | *B*. *velezensis* | |
| Y4 | | leaf | | OQ382878 | | OQ408182 | | OQ408207 | | OQ408232 | | *B*. *velezensis* | |
| Y5 | | leaf | | OQ382879 | | OQ408183 | | OQ408208 | | OQ408233 | | *B*. *velezensis* | |
| Y6 | | leaf | | OQ382880 | | OQ408184 | | OQ408209 | | OQ408234 | | *B*. *velezensis* | |
| YB1 | | leaf | | OQ410271 | |  | | OQ578987 | |  | | *Microbacterium phyllosphaerae* | |
| H16 | | flower | | OQ410272 | |  | | OQ578988 | |  | | *Pseudomonas monsensis* | |

Supplemental Table 5 The disease prevention efficacy of the 27 bacteria under the greenhouse

| Strain | Species | Diameter of lesion（mm） | Prevention efficacy（%） |
| --- | --- | --- | --- |
| CK |  | 12.1±3.5ab |  |
| AH7 | *B*. *velezensis* | 4.6±1.7efghij | 62.2±13.9cdefgh |
| AH8 | *B*. *velezensis* | 3.8±0.3fghijk | 68.9±2.3bcdef |
| H11 | *B*. *velezensis* | 3.8±1.1fghijk | 69.1±8.7bcdef |
| JB4 | *B*. *velezensis* | 4.6±1.0efghij | 62.3±8.0cdefgh |
| **XYAH1** | ***B***. ***velezensis*** | **0.2±0.2k** | **98.4±1.6a** |
| XYAH2 | *B*. *velezensis* | 6.0±1.2cdefgh | 51.0±10.0efghij |
| XYBH7 | *B*. *velezensis* | 4.5±2.1efghij | 63.2±14.9cdefgh |
| XYCH7 | *B*. *velezensis* | 7.0±1.0cdef | 42.1±8.5ghijk |
| **XYCJ12** | ***B***. ***velezensis*** | **3.4±0.3ghijk** | **72.2±2.2abcde** |
| XYDJ1 | *B*. *velezensis* | 7.5±1.8cde | 37.9±14.5hijk |
| XYDJ2 | *B*. *velezensis* | 5.0±0.9efghi | 58.6±7.5cdefgh |
| Y4 | *B*. *velezensis* | 8.9±1.3bcd | 26.4±10.9jk |
| Y5 | *B*. *velezensis* | 8.8±0.9bcd | 27.5±7.6ijk |
| Y6 | *B*. *velezensis* | 4.7±0.3efghi | 61.4±2.5cdefgh |
| AJ5 | *B*. *subtilis* | 14.2±1.1a | -16.9±9.3l |
| AJ8 | *B*. *subtilis* | 6.9±0.6cdefg | 43.2±5.3fghijk |
| AJ9 | *B*. *subtilis* | 7.2±1.7cdef | 40.6±13.9ghijk |
| **XYAJ8** | ***B***. ***subtilis*** | **3.8±1.5fghijk** | **68.6±12.5bcdef** |
| AY13 | *B*. *subtilis* | 5.6±1.1defgh | 53.8±9.1defghi |
| **AJ4** | ***B***. ***subtilis*** | **1.9±0.7ijk** | **84.2±5.7abc** |
| AY12 | *B*. *subtilis* | 9.3±1.3bc | 23.1±10.7k |
| XYBJ13 | *B*. *subtilis* | 6.8±0.9cdefg | 44.1±8.0fghijk |
| JB1 | *B*. *subtilis* | 4.2±0.5efghij | 65.6±4.2cdefg |
| **J1** | ***B***. ***altitudinis*** | **5.5±1.3defghi** | **54.6±10.7defgh** |
| **XYDH11** | ***B***. ***creceus*** | **3.8±0.6fghijk** | **68.6±4.6bcdef** |
| **YB1** | ***M***. ***phyllosphaerae*** | **2.5±1.5hijk** | **79.6±12.4abcd** |
| **H16** | ***P***. ***monsensis*** | **1.0±0.3jk** | **92.0±2.5ab** |

Note: Values represent means ± standard error from three replicates, means in each column with the same letters are not significantly different (P<0.05). The bold strains were selected as representative strains for subsequent tests.

Supplemental Table 6 Growth of the eight bacterial strains in response to salt with different concentration

| Strain | species | 2% | 5% | 7% | 10% |
| --- | --- | --- | --- | --- | --- |
| XYAH1 | *B*. *velezensis* | + | + | + | + |
| XYCJ12 | *B*. *velezensis* | + | + | + | + |
| XYAJ8 | *B*. *subtilis* | + | + | + | + |
| AJ4 | *B*. *subtilis* | + | + | + | + |
| XYDH11 | *B*. *cereus* | + | + | + | + |
| J1 | *B*. *altitudinis* | + | + | + | + |
| H16 | *P*. *monsensis* | + | + | - | - |
| YB1 | *M*. *phyllosphaerae* | + | + | + | - |

Note: “＋” indicated positive, “－” indicated negative.

Supplemental Table 7 Results of physiological and biochemical characterization

| characteristics | *B. velezensis* | | *B. subtilis* | | *B. cereus* | *B. altitudinis* | *P.monsensis* | *M. phyllosphaerae* |
| --- | --- | --- | --- | --- | --- | --- | --- | --- |
|  | XYAH1 | XYCJ12 | XYAJ8 | AJ4 | XYDH11 | J1 | H16 | YB1 |
| Citrate utilization | + | + | + | + | + | + | + | + |
| Contact enzyme reaction | + | + | + | + | + | + | + | + |
| Methyl red reaction | - | - | - | - | - | + | + | + |
| V-P reaction | + | + | + | + | + | - | - | - |
| Starch hydrolysis | + | + | + | + | + | + | - | + |
| Gelatin hydrolysis | + | + | + | + | + | + | + | + |
| Cellulose hydrolysis | + | + | + | + | + | + | + | + |
| a-lactose utilization | - | - | - | - | - | - | - | + |
| D-maltose utilization | + | + | + | + | + | - | + | + |
| D-sorbitol utilization | + | + | + | + | + | - | - | - |
| D-xylose utilization | + | + | + | + | - | - | - | + |
| Inositol utilization | + | + | + | + | + | - | + | + |
| D-mannitol utilization | + | + | + | + | - | + | + | + |
| D-fructose utilization | + | + | + | + | + | + | + | - |
| Sucrose utilization | + | + | + | + | + | + | + | + |
| D-glucose utilization | + | + | + | + | + | + | + | - |
| D-galactose utilization | - | - | - | - | - | - | - | - |
| Dulcitol utilization | - | - | - | - | - | - | - | - |
| L- rhamnose utilization | - | - | - | - | - | - | - | - |

Note: “＋” indicated positive, “－” indicated negative.

Supplemental Table 8 Biocontrol efficacy of the representative bacterial isolates against anthracnose of pearl plum under the greenhouse conditions.

| Representatives | | Disease severity index (%) | | Incidence rate（%） | | Control efficacy（%） | |
| --- | --- | --- | --- | --- | --- | --- | --- |
|  |  | Pre | Tre | Pre | Tre | Pre | Tre |
| CK |  | 46.7±2.9a | 59.3±4.1a | 68.7±2.0a | 86.7±3.3a |  |  |
| *B*. *velezensis* XYAH1 | | 2.6±1.6bc | 0.7±0.7d | 11.1±6.4bc | 3.7±3.7f | 94.4±3.5ab | 98.8±1.0a |
| *B*. *velezensis* XYCJ12 | | 4.2±0.6bc | 14.2±3.6c | 7.0±1.8bc | 41.7±2.1c | 90.9±1.3b | 76.1±6.1b |
| *B*. *subtilis* XYAJ8 | | 0.0±0.0c | 12.1±2.6c | 0.0±0.0c | 36.4±0.0cd | 100.0±0.0a | 79.6±4.5b |
| *B*. *subtilis* AJ4 | | 6.3±2.5b | 15.9±0.8c | 14.6±5.5b | 31.8±1.6de | 86.5±5.4b | 73.3±1.4b |
| *B*. *receus* XYDH11 | | 4.9±1.1b | 11.8±1.6c | 10.6±1.5bc | 25.4±1.6e | 89.5±2.4ab | 80.2±2.7b |
| *B*. *altitudinis* J1 | | 0.0±0.0c | 12.4±2.7c | 0.0±0.0c | 26.2±2.4e | 100.0±0.0a | 79.1±4.5b |
| *P*. *monsensis* H16 | | 3.8±1.4bc | 12.1±1.2c | 12.9±5.7b | 39.4±3.0c | 91.7±3.1ab | 79.6±2.0b |
| *M*. *phyllosphaerae* YB1 | | 6.2±1.5b | 34.4±2.7b | 18.0±5.1b | 62.4±1.2b | 86.6±3.3b | 42.0±4.6c |

Note: Values represent means ± standard error from three replicates, means in each column with the same letters are not significantly different (P<0.05). Pre: pre-treatment, Tre: treatment.

Supplemental Table 9 Effects of different medium on antagonistic activity of bacterial strains

| Inhibition rate（%） | | | | | | | | |
| --- | --- | --- | --- | --- | --- | --- | --- | --- |
| Media | YB1 | H16 | J1 | AJ4 | XYCJ12 | XYDH11 | XYAJ8 | XYAH1 |
| LB | 53.1±2.6a | 69.9±11.5a | 45.0±12.1a | 31.2±2.7b | 26.0±3.3ab | 35.5±9.8a | 42.2±7.5a | 30.8±4.5ab |
| PSA | 31.8±2.2b | 32.5±9.3a | 37.4±15.0a | 65.6±10.7a | 11.0±1.1b | 20.4±3.5a | 25.2±11.3a | 26.9±11.4ab |
| NA | 34.4±10.5ab | 56.8±15.8a | 31.2±16.7a | 45.4±1.4b | 16.3±1.9b | 28.4±3.2a | 30.8±11.5a | 24.5±8.1b |
| PDA | 10.3±3.3c | 48.0±13.7a | 17.2±2.6a | 28.8±1.4b | 38.9±11.8a | 29.2±12.6a | 44.7±3.7a | 53.5±8.6a |

Note: values represent means ± standard error from three replicates, means in each column with the same letters are not significantly different (P<0.05).

Supplemental Table 10 Chemical composition of VOCs produced by bacterial isolates with antifungal activity, analyzed by HS-SPME/GC-MS

|  |  |  |  |  |  |  |  |  |
| --- | --- | --- | --- | --- | --- | --- | --- | --- |
| **Bacterial isolates** | | | | | **Compounds** | **RT(min)** | **RA（%）** | **S(%)** |
| *B*. *subtilis*  XYAJ8 | | | | | **2-Heptanone** | **6.002** | **15.45059344** | **90** |
|  |  |  |  |  | **6-Methyl-2-Heptanone** | **8.582** | **6.887790063** | **94** |
|  |  |  |  |  | **5-Methyl-2-Heptanone** | **8.979** | **7.77096274** | **93** |
|  |  |  |  |  | D-Limonene | 11.483 | 4.424464359 | 95 |
|  |  |  |  |  | Pentacosane | 21.49 | 2.566643217 | 91 |
|  |  |  |  |  | Isopropyl octadecyl ether | 28.847 | 1.085438787 | 90 |
|  |  |  |  |  | Butylated Hydroxytoluene | 29.154 | 2.321704582 | 92 |
|  |  |  |  |  | 3,5-bis(1,1-dimethylethyl)- Phenol | 29.27 | 0.714738911 | 91 |
|  |  |  |  |  | Pentadecafluorooctanoic acid, octadecyl ester | 31.372 | 2.522258317 | 91 |
| *B*. *subtilis* AJ4 | | | | | **Dimethyl-Silanediol** | **3.794** | **21.1517583** | **91** |
|  |  |  |  |  | 2,5-dimethyl-Pyrazine | 6.51 | 0.51954941 | 93 |
|  |  |  |  |  | **Methoxy-phenyl-Oxime** | **8.602** | **44.2233854** | **90** |
|  |  |  |  |  | D-Limonene | 11.498 | 0.78177699 | 95 |
|  |  |  |  |  | 2-Ethyl-3,5-dimethyl-Pyrazine | 13.515 | 1.04070683 | 94 |
|  |  |  |  |  | Dodecyl nonyl ether | 23.064 | 0.38437636 | 90 |
|  |  |  |  |  | Tetradecane | 25.156 | 1.57885532 | 98 |
|  |  |  |  |  | Pentadecane | 28.983 | 1.28632716 | 97 |
|  |  |  |  |  | Butylated Hydroxytoluene | 29.154 | 0.49190937 | 92 |
|  |  |  |  |  | 11,20-didecyl- Triacontane | 33.378 | 0.22919191 | 91 |
|  | | | | |  |  |  |  |
| *B*. *velezensis*  XYAH1 | | | | | **2-Heptanone** | **6.037** | **13.92387941** | **94** |
|  |  |  |  |  | **6-Methyl-2-Heptanone** | **8.587** | **25.37996418** | **95** |
|  |  |  |  |  | D-Limonene | 11.488 | 0.634986181 | 95 |
|  |  |  |  |  | 2-Nonanone | 14.158 | 4.817361618 | 95 |
|  |  |  |  |  | 2-Decanone | 16.653 | 2.763937997 | 90 |
|  |  |  |  |  | 2-Dodecanone | 23.678 | 3.047614827 | 93 |
|  |  |  |  |  | 2-Tridecanone | 28.701 | 3.053974568 | 96 |
|  |  |  |  |  | 2-Tetradecanone | 30.748 | 3.459155791 | 91 |
|  |  |  |  |  | 2-Methyl-Z-4-tetradecene | 32.729 | 0.238523904 | 90 |
|  |  |  |  |  | 2-Hexadecanone | 34.132 | 3.172661462 | 96 |
|  |  |  |  |  | 2-Heptadecanone | 35.842 | 0.277039271 | 90 |
|  | | | | |  |  |  |  |
| *B*. *velezensis* XYCJ12 | | | | | **2-Heptanone** | **6.027** | **13.33329947** | **94** |
|  |  |  |  |  | **6-Methyl-2-Heptanone** | **8.582** | **8.337522094** | **95** |
|  |  |  |  |  | 2-Nonanone | 14.159 | 3.004682134 | 97 |
|  |  |  |  |  | 2-Decanone | 16.648 | 1.721751809 | 90 |
|  |  |  |  |  | 2-Undecanone | 21.531 | 2.773170041 | 94 |
|  |  |  |  |  | 3-ethyl-5-(2-ethylbutyl)-Octadecane | 23.069 | 0.445916567 | 90 |
|  |  |  |  |  | 2-Dodecanone | 23.678 | 2.510556438 | 95 |
|  |  |  |  |  | 2-Tridecanone | 28.706 | 6.960329918 | 97 |
|  |  |  |  |  | 2-Tetradecanone | 30.577 | 4.743435301 | 93 |
|  |  |  |  |  | 2-Hexadecanone | 34.132 | 5.80235611 | 95 |
| *B*. *receus* XYDH11 | | | | | D-Limonene | 11.503 | 2.226866226 | 95 |
|  |  |  |  |  | Benzyl alcohol | 11.755 | 4.615044303 | 96 |
|  |  |  |  |  | **4-Ethyl-decane** | **12.937** | **18.81350002** | **90** |
|  |  |  |  |  | Pentacosane | 21.214 | 6.211191351 | 90 |
|  |  |  |  |  | 1-Decene | 27.861 | 7.334701829 | 93 |
|  |  |  |  |  | Butylated Hydroxytoluene | 29.154 | 1.39709231 | 96 |
|  |  |  |  |  | Cyclopentadecane | 32.875 | 1.163081027 | 99 |
|  |  |  |  |  | 1,2-Benzenedicarboxylic acid, bis(2-methylpropyl) ester | 35.399 | 0.710799895 | 90 |
|  |  |  |  |  | Dibutyl phthalate | 36.45 | 1.18502866 | 91 |
|  | | | | |  |  |  |  |
| *B*. *altitudinis* J1 | | | | | **6-Methyl-2-Heptanone** | **8.602** | **10.54478241** | **95** |
|  |  |  |  |  | D-Limonene | 11.473 | 3.014529095 | 95 |
|  |  |  |  |  | **4-Ethyl-decane** | **13.143** | **9.00459371** | **90** |
|  |  |  |  |  | 2-Methyl-Z-4-tetradecene | 32.729 | 1.972691752 | 96 |
|  |  |  |  |  | 2-Hexadecanone | 34.132 | 2.647606266 | 93 |
|  |  |  |  |  | Dibutyl phthalate | 36.451 | 2.559844571 | 96 |
|  | | | | |  |  |  |  |
| *P*. *monsensis* H16 | | | | | 2-Heptanone | 6.072 | 0.612049919 | 94 |
|  |  |  |  |  | p-Cresol | 13.862 | 0.282309066 | 97 |
|  |  |  |  |  | **2-Nonanone** | **14.244** | **15.66703981** | **94** |
|  |  |  |  |  | 1-Nonene | 14.294 | 3.751391212 | 92 |
|  |  |  |  |  | 2-Decanone | 17.98 | 0.595563192 | 97 |
|  |  |  |  |  | **2-Undecanone** | **21.631** | **14.67946237** | **95** |
|  |  |  |  |  | 2-Undecanol | 21.882 | 2.161137263 | 90 |
|  |  |  |  |  | 2-Dodecanone | 24.874 | 0.588297604 | 96 |
|  |  |  |  |  | **2-Tridecanone** | **28.822** | **16.55186978** | **98** |
|  |  |  |  |  | 2-Heptadecanol | 29.033 | 3.678798406 | 91 |
|  |  |  |  |  | Butylated hydroxytoluene | 29.194 | 0.094006013 | 90 |
|  |  |  |  |  | 4-(4-amino-3-furazanylazo)- phenol | 31.552 | 0.895590605 | 95 |
|  |  |  |  |  | Z-8-Tetradecen-1-yl acetate | 32.966 | 1.040627757 | 90 |
|  |  |  |  |  | 2-Pentadecanone | 33.222 | 4.666624527 | 96 |
|  |  |  |  |  | Tricosan-2-ol | 33.307 | 0.184378642 | 90 |
|  |  |  |  |  | 2-Heptadecanone | 35.842 | 1.365305175 | 98 |
|  | | | | |  |  |  |  |
| *M*. *phyllosphaerae* YB1 | | | | | **Benzyl alcohol** | **11.72** | **20.6857732** | **98** |
|  |  |  |  |  | **4-Ethyl-decane** | **12.942** | **20.591619** | **90** |
|  |  |  |  |  | **Phenylethyl alcohol** | **14.732** | **12.04383** | **91** |
|  |  |  |  |  | Benzyl methyl ketone | 15.28 | 2.31681856 | 95 |
|  |  |  |  |  | Nonadecane | 21.495 | 1.35434056 | 91 |
|  |  |  |  |  | (E)-3-Eicosene | 28.691 | 1.18556827 | 95 |
|  |  |  |  |  | **Cetene** | **30.552** | **9.82244389** | **95** |
|  |  |  |  |  | 2-Heptadecanone | 35.842 | 0.75423856 | 94 |
|  |  |  |  |  | Dibutyl phthalate | 36.456 | 0.39586415 | 90 |

Note: Volatile organic compounds were extracted by SPME and identified through GC-MS. Forty-seven in total identified compounds and their retention times in GC chromatogram were listed in the table. The compounds with relatively high concentration were in bold text. RT represents the retention times in minutes. RA represents the relative peak area (relative area concentration) of the different compounds detected for each bacterial isolate, expressed as a percentage. S(%) = Similarity percentage.
